# Engineered RAP-anchored copper-escorting liposomes for FDX1-targeted cuproptosis in glioblastoma therapy‌

**DOI:** 10.7150/thno.115723

**Published:** 2025-07-02

**Authors:** Meng-Meng Liu, Ling-Xiao Zhao, Zhi-Qiang Gong, Yi-Jiang He, Xue Jiang, Wei Luo, Xin Yu, Zhan-You Wang

**Affiliations:** 1 Key Laboratory of Medical Cell Biology of Ministry of Education, Key Laboratory of Major Chronic Diseases of Nervous System of Liaoning Province, Health Sciences Institute of China Medical University, 110122, Shenyang, China.; 2 Cancer Hospital of China Medical University, Cancer Hospital of Dalian University of Technology, Liaoning Cancer Hospital & Institute, 110042, Shenyang, China.; 3 Department of Rehabilitation, Shengjing Hospital of China Medical University, 110134, Shenyang, China.; 4 State Key Laboratory of Quantitative Synthetic Biology, Shenzhen Institute of Synthetic Biology, Shenzhen Institutes of Advanced Technology, Chinese Academy of Sciences, 518055, Shenzhen, China.

**Keywords:** elesclomol, copper, glioblastoma, cuproptosis, receptor-associated protein

## Abstract

**Rationale:** Glioblastoma multiforme (GBM), the most aggressive primary brain malignancy, presents considerable therapeutic challenges due to intrinsic treatment resistance and dismal clinical outcomes. Capitalizing on emerging insights into cuproptosis-mediated oncotherapy, we have developed a receptor-associated protein (RAP)-modified liposomal nanoplatform (RAP-LPs@ESCu) for the precise delivery of elesclomol-copper complexes (ESCu) and aimed to evaluate its therapeutic potential in triggering tumor-specific cuproptosis.

**Methods:** RAP-LPs@ESCu were synthesized via thin-film hydration and characterized by transmission electron microscope (TEM) and dynamic light scatting. Intracellular copper levels were quantified via atomic absorption spectroscopy. RNA sequencing was used to identify cuproptosis-related molecular targets, among which ferredoxin-1 (FDX1) was the primary focus of the study. Western blot, immunohistochemistry, immunofluorescence, flow cytometry and biochemical kits were applied to elucidate the molecular mechanism of cuproptosis triggered by RAP-LPs@ESCu. Orthotopic GBM models were established by stereotactic implantation of luciferase-labeled LN229 cells into the right striatum of BALB/c-nu mice. In vivo imaging system was utilized to monitor tumor progression and blood-brain barrier (BBB) penetration. Copper content in tumor tissues was quantified by biochemical kit, and mitochondrial morphology was examined by TEM. Systemic toxicity of RAP-LPs@ESCu was evaluated through hematological, biochemical, hemolysis assays, and hematoxylin-eosin staining. Neurological and motor functions were assessed using the Loga 5-score test and open-field test.

**Results:** Through systematic evaluation in an orthotopic xenograft mouse model, we found that RAP-LPs@ESCu effectively induced cuproptosis, inhibited GBM progression, and significantly prolonged survival. Mechanistic studies revealed that RAP-mediated targeting resulted in efficient BBB penetration and preferential accumulation of ESCu in tumor cells. Subsequent intracellular Cu²⁺ overload triggered a cascade of molecular events beginning with substantial upregulation of FDX1 expression, followed by accumulation of lipoylated dihydrolipoamide S-acetyltransferase aggregates, and finally depletion of iron-sulfur cluster proteins. These coordinated effects culminated in the selective induction of cuproptosis in GBM cells.

**Conclusions:** This study successfully constructed RAP-LPs@ESCu which selectively eliminated mitochondria-metabolically active GBM cells via an FDX1-dependent cuproptosis pathway, ultimately achieving orthotopic GBM growth suppression.

## Introduction

Glioblastoma multiforme (GBM), classified as a World Health Organization (WHO) grade IV glioma, represents the most aggressive primary brain malignancy characterized by inevitable recurrence, neuroparenchymal infiltration, and profound therapeutic resistance 1, 2. While chemotherapy constitutes a mainstay therapeutic approach, its clinical efficacy remains severely limited by the blood-brain barrier (BBB), intratumoral heterogeneity, and acquired drug resistance 3, 4. These challenges have spurred significant innovation in nanomedicine, where advanced delivery systems demonstrate enhanced therapeutic precision through controlled pharmacokinetics and molecular targeting 4.

Liposomes (LPs) have been widely employed as drug carriers for brain tumor therapy, due to their high drug-loading capacity, excellent biocompatibility, controlled release properties, non-toxicity and non-immunogenicity 5. However, conventional LPs face pharmacokinetic limitations, including rapid mononuclear phagocyte system clearance and passive tumor accumulation via enhanced permeability and retention effects often resulting in subtherapeutic drug levels 6. Polyethylene glycol (PEG) conjugation extends systemic circulation through steric stabilization, while ligand-mediated active targeting enables preferential accumulation in neoplastic tissues through receptor-ligand interactions 7. Notably, low-density lipoprotein receptor-related protein-1 (LRP1) serves as a dual-target biomarker, as it is highly expressed on cell membrane of GBM as well as on the endothelial cell membrane of the BBB, the blood-tumor barrier (BTB), and the tumor-associated neovasculature 8, 9. Receptor-associated protein (RAP), a 39 kDa molecular chaperone, exhibits high-affinity LRP1 binding and has been successfully employed to develop brain-penetrant nanodelivery system 10. These studies inspired us to develop RAP-modified liposome-based nanodrugs (RAP-LPs) as a strategy against GBM.

An increasing number of studies have highlighted the therapeutic potential of targeting the cuproptosis of tumor cells as a novel anti-cancer strategy, including GBM 11-15. Cuproptosis is a novel form of copper overload-induced cell death, primarily characterized by the accumulation of lipoylated proteins and the depletion of iron-sulfur (Fe-S) clusters 16. Ferredoxin-1 (FDX1) plays a crucial role as a key upstream regulator of cuproptosis 17. FDX1 regulates the lipoylation of dihydrolipoamide S-acetyltransferase (DLAT) and facilitates the reduction of Cu²⁺ to Cu⁺, which directly binds to lipoylated DLAT. This interaction induces the polymerization of lipoylated DLAT and leads to the loss of Fe-S clusters, which together trigger proteotoxic stress and cell death 18, 19. Interestingly, elesclomol (ES) can exert anti-cancer effects by modulating FDX1 20. ES is a Cu ionophore that forms redox-active complexes (ESCu), which selectively deliver Cu²⁺ to mitochondria 21. Recent studies have shown that ES is able to induce cuproptosis in liver cancer cells through elevation of intracellular copper levels 22. Given the limited exploration of cuproptosis in GBM, we sought to investigate whether the induction of cuproptosis could be achieved by enhancing the delivery of ESCu to GBM cells, and to explore the mechanistic pathways involved.

In this study, we constructed a brain-targeted liposome loaded with ESCu (RAP-LPs@ESCu), which integrates a precise drug delivery system with the cuproptosis susceptibility of GBM cells. The results demonstrated that RAP-LPs@ESCu efficiently delivered ESCu to GBM cells, resulting in an increased intracellular Cu²⁺ levels, upregulated FDX1 expression, and ultimately induced cuproptosis. Moreover, in an orthotopic GBM xenograft mouse model, RAP-LPs@ESCu exhibited superior tumor growth suppression and survival prolongation compared to non-targeted counterparts. These findings highlight RAP-LPs@ESCu as a promising strategy for GBM treatment through the targeted induction of cuproptosis.

## Materials and Methods

### Cell lines and culture

Human GBM cell lines LN229 and T98G were obtained from the Cell Bank of the Chinese Academy of Sciences (Shanghai), and human cerebral microvascular endothelial cells (hCMEC/D3) were obtained from Pricella (Wuhan). All cells were cultured in DMEM (Biological Industries) supplemented with 10% fetal bovine serum and 1% penicillin/streptomycin at 37 °C with 5% CO_2_. For *in vitro* experiments, cells were treated with ESCu (100 nM), LPs@ESCu (400 nM, equivalent ESCu concentration 100 nM), or RAP-LPs@ESCu (400 nM, equivalent ESCu concentration 100 nM) for 24 h. Tetrathiomolybdate (TTM) (SJ-MK6196A, Spakjade) was used at a concentration of 10 μM for 24 h.

### Preparation of liposomes

DSPE-PEG_2000_-RAP was synthesized based on previous studies 23. Specifically, DSPE-PEG_2000_-MAL (190 mg, Xarxbio) and RAP13 peptide (80 mg, Sangon Biotech) were dissolved in 40 mL of 10% aqueous acetic acid, and the mixture was stirred under a nitrogen atmosphere for 4 h at room temperature. The resulting solution was dialyzed using a 2 kDa dialysis bag for 24 h at room temperature to obtain the tumor-targeting polymer DSPE-PEG_2000_-RAP. The structure of the final product was determined by ^1^H NMR spectroscopy in DMSO at 600 MHz. The product was then freeze-dried and stored at 4 °C.

Liposomes were prepared using the conventional thin film hydration method 24. Briefly, egg yolk lecithin (EPC) (100 mg, S30871, Genye), cholesterol (30 mg, C6213, Macklin), DSPE-MPEG_2000_ (4 mg, D875844, Macklin) and DSPE-PEG_2000_-RAP (0.2 mg) were dissolved in 5 mL of a mixture of methanol and chloroform (molar ratio, 1/1). The solution was then evaporated for 1 h in a water bath at 48 °C and 95 r/min under reduced pressure to form a homogeneous and transparent film. Subsequently, the derived thin film was hydrated in 5 mL PBS (pH 7.4) at 50 °C for 30 min, followed by sonication in an ice-water bath at 200 W for 10 min. The blank liposomes were obtained by filtration through a 0.22 μm aseptic member and stored at 4 °C. Similarly, other group formulations of liposomes can be obtained by incorporating an ESCu (3 mg), RAP (molar ratio, 2.5%), DiR (0.1 mg, HY-D1048, Med Chem Express) and Coumarin 6 (Cou6) (0.5 mg, C7810, Solarbio) into the 5 mL of the mixed organic solution. Moreover, the ESCu complexes were prepared using a 1:1 molar ratio of elesclomol (ES) (HY-12040, Med Chem Express) to Cu^2+^. X-ray photoelectron spectroscopy (XPS) analysis was performed to determine the valence state of copper in ESCu.

### Characterization of liposomes

The morphology of liposomes was observed using transmission electron microscope (TEM) (TecnaiG2, FEI). The hydrodynamic diameter and zeta potential of the liposomes were measured using dynamic light scatting (DLS) (Zetasizer Nano ZS). The morphology and elemental composition of the liposomes were characterized using field emission scanning electron microscopy (FESEM) (Clara, Tescan).

### ESCu loading

ESCu was first scanned across a full wavelength range to determine its optimum absorption wavelength (258 nm) using UV spectrophotometry, which was used to generate its standard curve (*y = 0.0503x + 0.2913, R^2^ = 0.9932*). Different liposomes were prepared according to mass ratios (ES/EPC) of 1:100, 2:100, 3:100, 4:100, and 5:100. RAP-LPs@ESCu (100 μL) was mixed with methanol (100 μL) and then subjected to ultrasonic emulsification (300 W). The mixture was then centrifuged at 12,000 rpm for 30 min at 4 °C, and the supernatant was collected for measurement. The encapsulation efficiency (EE%) was calculated using the following equations.

EE% = Weight of ESCu in RAP-LPS@ESCu/Weight of the feeding ESCu × 100%

### Cell viability assay

The CCK-8 assay (GK10001, Glpbio) was employed to evaluate cell viability. LN229, T98G, and hCMEC/D3 cells were seeded in 96-well plates and incubated at 37 °C in an incubator containing 5% CO₂ for 24 h. The cells were then incubated in serum-free medium for 6 h before receiving the indicated treatments. CCK-8 working solution was added for 1 h and the absorbance was detected at 450 nm with a UV-vis-NIR spectra reader (Cytation 5, Bio Tek). Concentration gradients were established based on ES: ES (0, 0.2, 0.4, 0.8, 1.0, 2.0 μM), ESCu (0, 0.05, 0.1, 0.2, 0.4, 0.8 μM), and liposomes (0, 0.1, 0.2, 0.4, 0.8, 1.0, 2.0 μM).

### Live/dead staining assay

The cytotoxic effects of RAP-LPs@ESCu on GBM cells were assessed using calcein AM and propidium iodide (PI) probes (C2015S, Beyotime). LN229 and T98G cells were inoculated in 96-well plates and divided into six groups: Control, ESCu (100 nM), LPs, RAP-LPs, LPs@ESCu (loading 400 nM ESCu), and RAP-LPs@ESCu (loading with 400 nM ESCu). After 24 h incubation with the designated treatments, the cells were treated with serum-free medium containing calcein AM (5 μM) and PI (2 μg/mL) for 30 min. Fluorescence images were then captured using a fluorescence microscope. Live cells exhibited green fluorescence, while dead cells displayed red fluorescence.

### Annexin V-FITC/PI staining

The damaging effect of RAP-LPs@ESCu on GBM cells was assessed using the Annexin V-FITC/PI double staining kit (KGA108, KeyGEN). LN229 and T98G cells were collected, and a mixture of Annexin V-FITC and propidium iodide (PI) was added to the cell suspension. After a 10-min incubation at room temperature in the dark, cells were washed, resuspended in PBS, and analyzed by flow cytometry (FCM) (FACSCelesta, BD). Cell death was determined by fluorescence: Annexin V-FITC positive/PI positive indicated cell death, while Annexin V-FITC negative/PI negative indicated live cells.

### Transwell assay

Transwell plates (8 µm pore size) were used to assess cell invasion. LN229 and T98G cells were inoculated into the upper chamber, and various treatment factors were added to the lower chamber. After incubation at 37 °C for 24 h, migrating cells on the lower surface were fixed with 4% paraformaldehyde and stained with crystal violet for 30 min, followed by washing with PBS. Images were acquired using a microscope.

### Uptake and *In vitro* BBB translocation

For the cellular uptake test of liposomes, LN229 and T98G cells were directly inoculated into 24-well plates, and Cou6-labeled LPs@ESCu and RAP-LPs@ESCu were subsequently added for co-cultivation. Fluorescence images were captured at different time points (2, 4, 8 h) to compare the changes in fluorescence intensity.

An *in vitro* blood-brain barrier (BBB) model was established using hCMEC/D3 cells in a transwell cell culture system. Briefly, hCMEC/D3 cells (1 × 10^5^ cells/well) were seeded into the upper chamber, while LN229 or T98G cells were placed in the lower chamber. Cou6-labeled LPs@ESCu (loading 400 nM ESCu) and RAP-LPs@ESCu (loading 400 nM ESCu) were then added to the upper chamber. After a co-cultivation period of 24 h, fluorescence images of LN229 or T98G cells were obtained using fluorescence microscopy, and the fluorescence intensities of the two groups were subsequently compared.

### Determination of Cu content

In the* in vitro* experiments, LN229 and T98G cell samples were collected according to specific subgroups and acidified with nitric acid. The copper content in the cell lysates was then determined using atomic absorption spectroscopy (AAS). In the *in vivo* experiments, a copper (Cu²⁺) colorimetric assay kit (E-BC-K300-M, Elabscience) was used to measure copper content in GBM tissues. Briefly, a homogenate of the GBM tissue was prepared in double-distilled water, and the supernatant was centrifuged for analysis. Add 230 µL of color developer to 15 µL of the GBM sample and incubate at 37 °C for 5 min. The absorbance at 580 nm was measured using a microplate reader.

### Intracellular oxidative levels test

The reactive oxygen species (ROS) content in cells was evaluated using the ROS assay kit (S0022M, Beyotime). For *in vitro* studies, LN229 and T98G cells were seeded in 6-well plates. After all the treatments, cells were collected and incubated with DCFH-DA probe at 37 °C for 30 min. Following resuspension in PBS, fluorescence intensity was analyzed using FCM. For fluorescence imaging, LN229 and T98G cells were seeded on 14 mm coverslips. DCFH-DA staining solutions were added, and cells were incubated at 37 °C for 30 min. The images were acquired using confocal laser scanning microscope (CLSM) (A1, Nikon). For* in vivo* studies, fresh GBM tissues were obtained and washed with pre-cooled PBS before incubation with enzyme digestion solution. The digestion was terminated with PBS, and the cells were collected by centrifugation at 500 g for 10 min. The cells were then resuspended in DCFH-DA working solution and incubated for 1 h at 37 °C. Fluorescence was detected at 488 nm by microplate reader (Cytation 5, Bio Tek).

The glutathione (GSH) assay kit (MAK517, Sigma-Aldrich) was employed to measure GSH levels. Cell and GBM tissue samples were collected and washed with pre-cooled PBS Tissue samples were homogenized using EDTA lysis buffer. The homogenate was then centrifuged at 10,000 g for 15 min at 4 °C, and the supernatant was collected for analysis. GSH levels were determined using the DTNB colorimetric assay, with optical density measured at 412 nm.

### Mitochondrial morphology and function detection

Mitochondrial morphology of LN229 and T98G cells was examined using TEM (HT7800/HT7700, Hitachi). Briefly, cell samples were fixed in 2.5% glutaraldehyde for 2 h. Following dehydration, cells were cut into ultrathin sections (60-80 nm), stained with uranyl acetate and lead citrate for 15 min, and placed on copper grids for TEM observation.

Mitochondrial membrane potential was assessed using the mitochondrial membrane potential assay kit (JC-10, CA1303, Solarbio). The JC-10 working solution was prepared with PBS and incubated at 37 °C for 20 min, followed by fluorescence imaging using CLSM. The monomeric form of JC-10 showed green fluorescence, while the aggregated form displayed red fluorescence. Membrane potential was assessed by calculating the fluorescence intensity ratio of aggregates (red) to monomers (green).

Intracellular ATP levels were measured using the ATP chemiluminescence assay kit (E-BC-F002, Elabscience). A total of 100 µL of supernatant from cell lysates was mixed with the ATP working solution and incubated at room temperature for 5 min before detecting chemiluminescence values.

### Western blot

Proteins from GBM cells and tissues were extracted using RIPA lysis buffer (P0012B, Beyotime) containing protease inhibitors (HY-K0010, Med Chem Express) and phosphatase inhibitors (HY-K0021, Med Chem Express). Equal amounts of samples were loaded onto SDS-PAGE gels and then transferred to polyvinylidene fluoride (PVDF) membranes (IPVH00010, Merck Millipore). Primary antibodies were applied and incubated overnight at 4 °C. After incubation of corresponding horseradish peroxidase (HRP)-conjugated antibody for 1 h at room temperature, images were captured using a gel imaging system (5200, Tanon). Primary antibodies used in this study included anti-FDX1 (A20895, ABclonal), anti-DLAT (A8814, ABclonal), anti-LIAS (DF14836, Affinity), anti-ACO2 (A4524, ABclonal) and anti-GAPDH (10494-1-AP, Proteintech).

### Immunofluorescence staining

LN229 and T98G cells were seeded in cell culture dishes. After applying the designated treatments, cells were fixed in 4% paraformaldehyde for 15 min, followed by permeabilization with 0.5% Triton X-100 for 25 min. Blocking was performed with 5% BSA for 30 min. The primary antibody was incubated overnight at 4 °C, and the samples were then treated with a fluorescent secondary antibody. Finally, the samples were stained with DAPI (C0060, Solarbio) for 5 min to visualize the cell nuclei. Images were acquired using a CLSM. Primary antibodies used in this study included anti-FDX1 (A20895, ABclonal) and anti-DLAT (CQA8293, Cohesion).

### Immunohistochemical staining

Paraffin-embedded mouse brain tissue sections (5 μm) were deparaffinized and subjected to antigen retrieval in sodium citrate buffer. Subsequent procedures were performed according to the instructions (SV0001, BOSTER) or (SV0002, BOSTER). Briefly, sections were treated with 3% H₂O₂ to inhibit endogenous peroxidase activity, followed by blocking with 5% BSA to minimize non-specific binding. The primary antibody was incubated overnight at 4 °C, followed by a 2 h incubation with biotinylated anti-mouse/rabbit IgG at room temperature**.** Color development was achieved with 3,3'-diaminobenzidine, followed by counterstaining of the nuclei with hematoxylin. Finally, the sections were subjected to gradient dehydration, and visualized with a light microscope (ECLIPSE Ci-L, Nikon). Primary antibodies used in this study included anti-FDX1 (A20895, ABclonal), anti-DLAT (CQA8293, Cohesion), ACO2 (A4524, ABclonal) and Ki67 (GB111499, Servicebio).

### Animals and orthotopic xenograft models

The 5-week-old pathogen-free female BALB/c-nu nude mice used in this study. Mice were fixed in a stereotaxic apparatus, and the skull was exposed. A three-dimensional coordinate system was established with the bregma as the origin. Luciferase-labeled LN229 (LN229-Luc) cell suspension (5 × 10^5^ cells) was injected at the following coordinates: anterior 0.5 mm, lateral 2 mm, and depth 3.5 mm. After 10 days, tumor growth was monitored and quantified using the *in vivo* imaging system (IVIS) (ABL X5, Tanon). The body weight and survival time of the mice were recorded.

All animals were approved by the Ethics Committee for Experimental Animal Welfare of China Medical University (CMU2023932).

### BBB translocation and biodistribution* in vivo*

Non-invasive near-infrared optical imaging technique was utilized to assess the brain-targeting capability of RAP-LPs@ESCu. The GBM model mice were randomly divided into three groups: DiR, DiR-LPs@ESCu, and DiR-RAP-LPs@ESCu. After intravenous injection, images were captured using IVIS at specified time intervals (1, 2, 4, 8, 12, 24 h). Regions of interest (ROIs) were delineated around the brain, and the fluorescence intensity of the DiR signal was analyzed using living image software. The mice were sacrificed at 24 h post-injection, and tumors alongside major organs (heart, liver, spleen, lung and kidney) were collected for fluorescence imaging. In addition, the orthotopic LN229-bearing nude mice were randomized into 3 groups receiving tail vein injections of ESCu, LPs@ESCu and RAP-LPs@ESCu, after 24 h treatment, major organs (heart, liver, spleen, lung and kidney) and GBM tissues were collected to quantify copper accumulation via inductively coupled plasma optical emission spectrometry (ICP-OES), expressed as percentage injected dose per gram of tissue (%ID/g).

### *In vivo* anti-tumor effects

Orthotopic nude mice bearing LN229-Luc xenograft tumors were established and randomly divided into 6 groups, (a) PBS, (b) ESCu (0.6 mg/mL, equivalent ES dose of 6 mg/Kg), (c) LPs, (d) RAP-LPs, (e) LPs@ESCu (0.6 mg/mL, equivalent ES dose of 6 mg/Kg), and (f) RAP-LPs@ESCu (0.6 mg/mL, equivalent ES dose of 6 mg/Kg). Mice were imaged using IVIS spectroscopy on day 10 to record brain bioluminescence. The mice were administered drug solutions intravenously and imaged every other day for a total of 6 times. Body weight and survival of the mice were monitored throughout the treatment schedule. On day 22, mice were euthanized and brains were isolated for paraffin sectioning. Glioma areas were observed using hematoxylin-eosin (H&E) staining.

### Statistical analysis

Statistical analyses utilized Student's *t*-test for two-group comparisons and one-way ANOVA for multiple groups. Survival curves were generated using the Kaplan-Meier method. Graphs were created and analyzed using ImageJ and GraphPad Prism 9 software. All experimental data are expressed as means ± standard errors (means ± SEM). *P*-value < 0.05 was considered statistically significant (*P < 0.05, **P < 0.01, ***P < 0.001).

## Results and Discussion

### Preparation and characterization of RAP-LPs@ESCu

**Figure 1:**
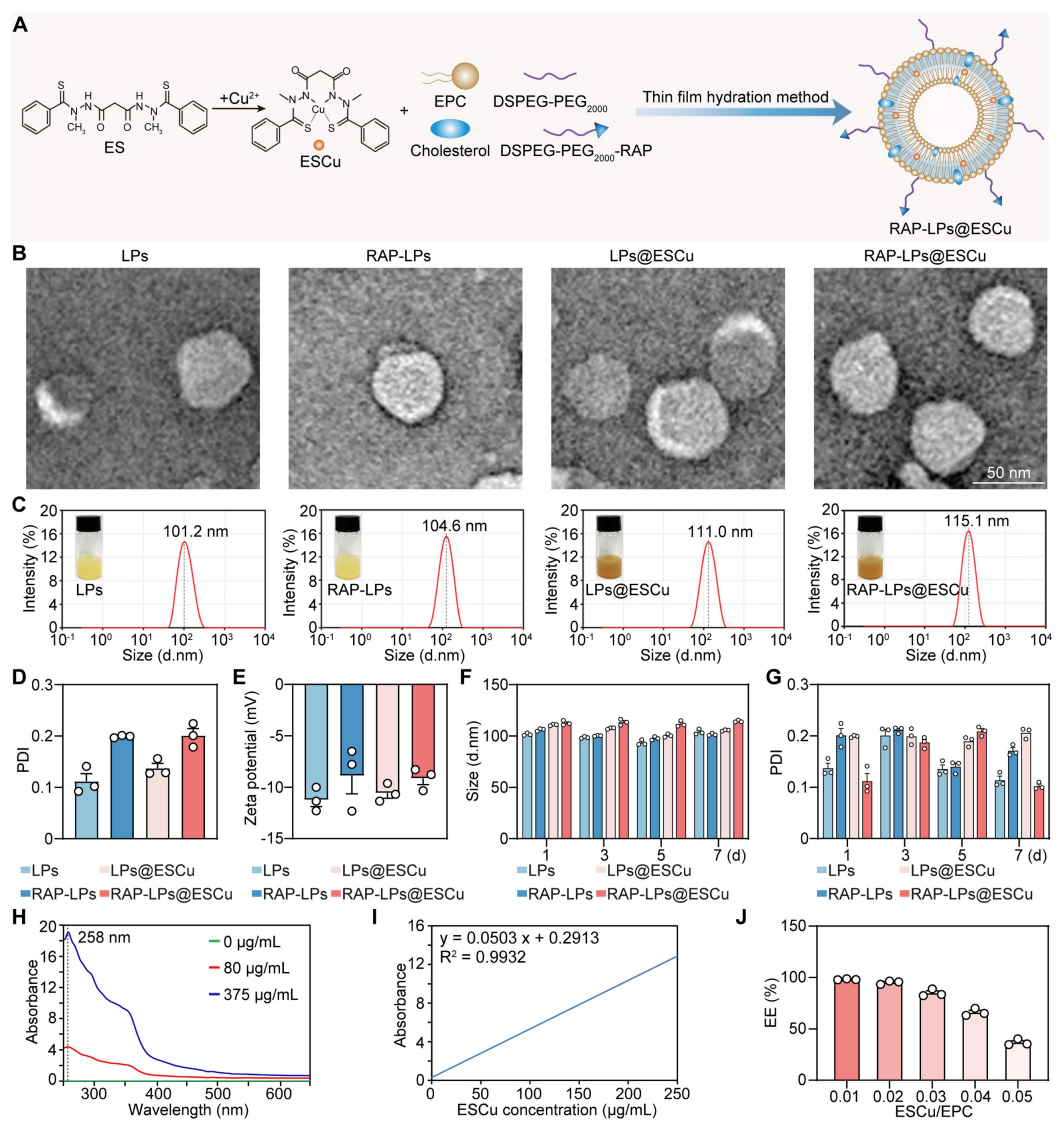
** Synthesis and characterization of RAP-LPs@ESCu.** (A) Schematic design of RAP-LPs@ESCu using thin film hydration method. (B) Representative TEM images of LPs, RAP-LPs, LPs@ESCu and RAP-LPs@ESCu. Scale bar, 50 nm. (C-E) Particle-size distribution, PDI and Zeta potentials of LPs, RAP-LPs, LPs@ESCu and RAP-LPs@ESCu obtained by DLS. (F-G) Time-dependent changes of size and PDI of LPs, RAP-LPs, LPs@ESCu and RAP-LPs@ESCu incubated in water at 4 ℃. (H) UV-Vis-NIR absorbance of different concentrations of ESCu showing absorption peak at 258 nm. (I) Standard curve of the UV-vis absorbance of ESCu with relative concentrations. (J) EE% of ESCu with different ESCu/EPC ratio. *n* = 3 independent biological replicates.

ES binds to Cu^2+^ to form a more active complex, ESCu. X-ray photoelectron spectroscopy (XPS) was employed to evaluate the Cu valences in the ESCu. The high-resolution Cu 2p XPS spectrum exhibited two characteristic Cu^2+^ (Cu-S) 2p peaks at Cu 2p_3/2_ (932.39 eV) and Cu 2p_1/2_ (951.74 eV) (Figure S3). This finding indicates that Cu valence in ESCu was +2. The therapeutic potential of ESCu is limited by rapid systemic clearance and insufficient tumor accumulation 25. To address these limitations, we leveraged the lipophilic nature of ESCu for liposomal encapsulation while incorporating LRP1-targeting functionality through RAP13 peptide conjugation, a strategy informed by BBB penetration studies 26. The nanoplatform was developed through sequential functionalization. As shown in Figure S1, a Michael addition reaction was used to link DSPE-PEG_2000_-MAL to the sulfhydryl group on cysteine in the RAP13 peptide, yielding DSPE-PEG_2000_-RAP 23. ^1^H NMR spectroscopy was conducted to determine the structural characteristics of DSPE-PEG_2000_-RAP. As shown in Figure S2, the spectrum of DSPE-PEG_2000_-MAL exhibited characteristic peaks at δ 0.850 ppm (peak a) and δ 1.228 ppm (peak b) for DSPE methyl and methylene protons; δ 3.505 ppm (peak c) for PEG protons and δ 7.017 ppm (peak d) for maleimide protons. The spectrum of DSPE-PEG_2000_-RAP retained the DSPE signals at δ 0.849 ppm (peak a) and δ 1.228 ppm (peak b) and the PEG signal at δ 3.505 ppm (peak c). Importantly, new signals attributed to the aromatic protons of the RAP peptide appeared at δ 6.976, δ 6.823 and δ 6.616 ppm, which confirmed the successful conjugation of the RAP peptide to DSPE-PEG_2000_-MAL. Subsequently, RAP-LPs@ESCu was prepared using the thin film hydration method with EPC, cholesterol, DSPE-PEG_2000_, DSPE-PER_2000_-RAP and ESCu (Figure 1A).

TEM images confirmed monodisperse spherical nanostructures with smooth surfaces, critical for prolonged vascular circulation (Figure 1B). To verify the successful loading of ESCu into liposomes, FESEM with elemental mapping was employed. The mapping analysis of RAP-LPs@ESCu group revealed distinct Cu signals, confirming ESCu incorporation (Figure S4B). Although FESEM revealed a significant particle size increase in both RAP-LPs and RAP-LPs@ESCu (Figure S4A), which might be primarily attributed to liposomal aggregation without cryoprotectants and FESEM's inherent edge-enhancement effect 27, 28, comparison with RAP-LPs controls showed markedly elevated Cu and S signals in RAP-LPs@ESCu. This confirms successful functionalization and indicates the size discrepancy does not compromise the study's core conclusions. Moreover, to directly visualize RAP incorporation, we performed SDS-PAGE with Coomassie blue staining. This analysis revealed distinct protein band for RAP-LPs, RAP-LPs@ESCu, and the positive control (RAP), which were absent in both plain LPs and LPs@ESCu, thereby confirming the successful conjugation of RAP to the LPs (Figure S5). Dynamic light scattering (DLS) showed that the hydrodynamic diameters of LPs, RAP-LPs, LPs@ESCu and RAP-LPs@ESCu were 101.2 nm, 104.6 nm, 111.0 nm and 115.1 nm, respectively (Figure 1C), and the polydispersity index (PDI) values were in the range of 0.1-0.3, which were well dispersed (Figure 1D). The zeta potentials of LPs, RAP-LPs, LPs@ESCu and RAP-LPs@ESCu were -11.22 ± 0.66 mV, -8.87 ± 1.76 mV, -10.56 ± 0.5 mV and -9.13 ± 0.622 mV, respectively (Figure 1E). No significant changes in particle size or PDI were observed over 7 days at 4 °C, indicating robust formulation integrity (Figure 1F-G). In addition, the optimum absorption wavelength for ESCu was determined to be 258 nm using UV spectrophotometry for EE% measurements (Figure 1H-I). As the ESCu/EPC ratio increased, the EE% decreased from 98.197 ± 0.241% to 36.79 ± 1.548% (Figure 1J). The ESCu/EPC ratio chosen for this study was 0.03, with an EE% of 84.93 ± 1.848%. Together with the images of the various components of the LPs, these results indicated that RAP-LPs@ESCu was successfully prepared.

### Cytotoxicity evaluation of RAP-LPs@ESCu

**Figure 2:**
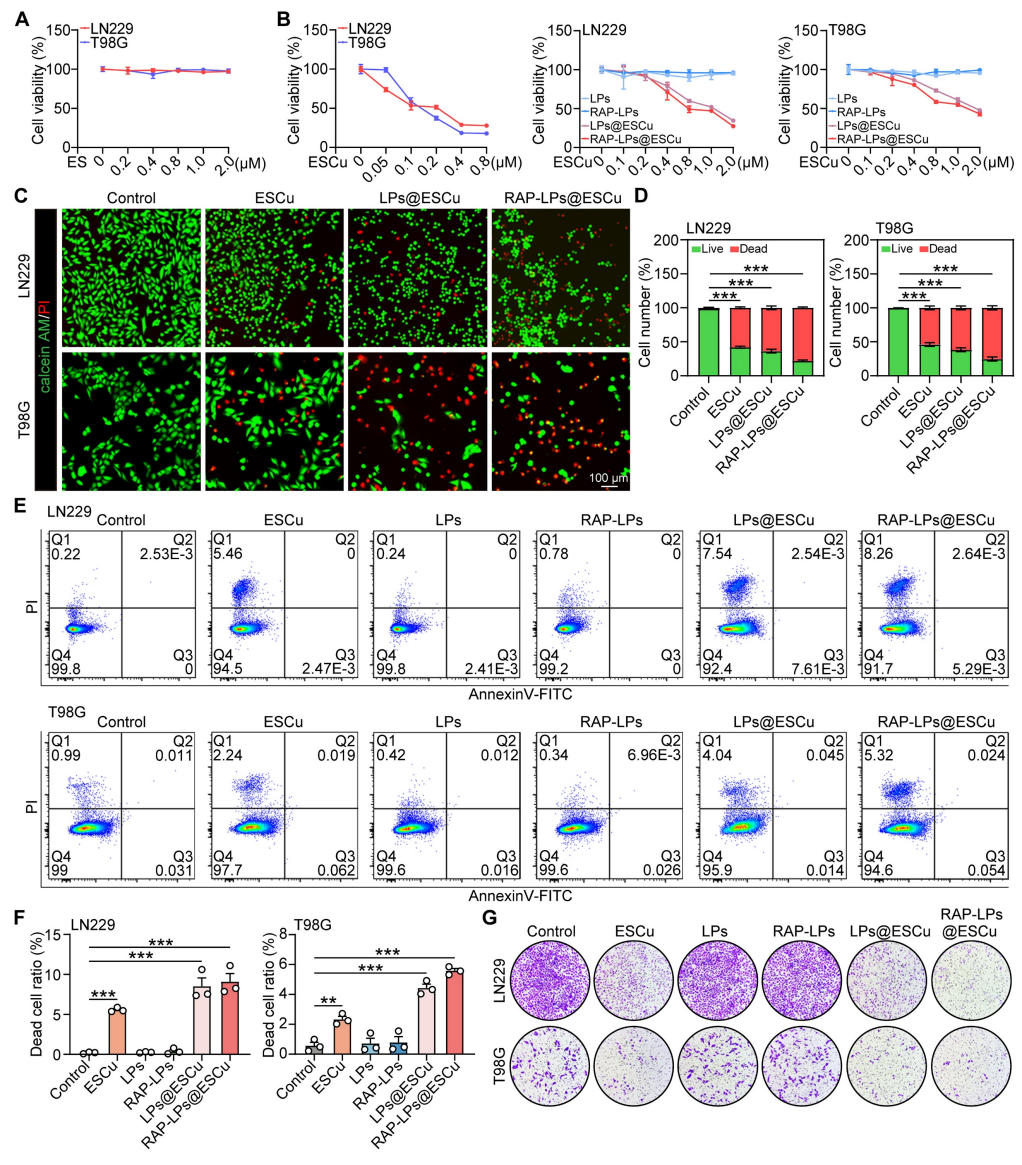
** Anti-GBM activity of RAP-LPs@ESCu *in vitro*.** (A) Cell viability of LN229 and T98G cells with ES. (B) Cell viability of LN229 and T98G cells with ESCu, LPs, RAP-LPs, LPs@ESCu and RAP-LPs@ESCu for 24 h. (C-D) Representative images of LN229 and T98G cells after different treatments, stained with calcein-AM and PI. Scale bar, 100 μm. (E-F) Following the indicated treatment, LN229 and T98G cells were stained with Annexin V-FITC and PI and subsequently analyzed by flow cytometry. (G) Transwell assay of LN229 and T98G cells treated with ESCu, LPs, RAP-LPs, LPs@ESCu and RAP-LPs@ESCu for 24 h. *n* = 3 independent biological replicates, 3 images were counted per group in each independent trial. **P < 0.01, ***P < 0.001.

To systematically evaluate the cytotoxic effects of RAP-LPs@ESCu on GBM cells, a series of *in vitro* assays were performed. It is noteworthy that ES at 2 μM (Figure 2A) and free copper ions at high concentrations (160 μM) did not exhibit significant toxicity (Figure S6), which may be limited by the content and activity of cotransporter proteins on the cells 29, 30. However, ESCu, LPs@ESCu and RAP-LPs@ESCu showed a concentration-dependent reduction in cell viability of LN229 and T98G cells in the 2 μM range (Figure 2B). Notably, RAP-LPs@ESCu exhibited a significantly higher half-maximal inhibitory concentration (IC50) than ESCu, probably due to its sustained drug release under acidic conditions (pH 5.0). A total of 77.563 ± 1.293% of ESCu was released from RAP-LPs@ESCu at pH 5.0 within 96 h, while the numerical value was decreased to 25.906 ± 1.316% at pH 7.4 (Figure S7). Liposomal systems undergo pH-dependent structural changes, with acidic environments enhancing membrane permeability and accelerating drug release 31. Importantly, the tumor microenvironment (TME) in GBM is intrinsically acidic due to factors such as the Warburg effect (aerobic glycolysis), hypoxia and poor perfusion 32, 33. This acidic gradient between the healthy circulation (pH 7.4) and acidic glioma TME provides a key endogenous trigger for the selective release of ESCu from the liposomes. However, their high encapsulation efficiency and stability enable controlled release, ensuring gradual drug diffusion, prolonged therapeutic effects, and optimized pharmacokinetics 34. Based on these results, ESCu (100 nM) and ESCu-loaded liposomal formulations (400 nM) were used in subsequent mechanistic investigations. Live/dead cell staining results showed a higher proportion of dead cells in the RAP-LPs@ESCu group compared to other groups (Figure 2C-D) (Figure S8A-B). The 5-ethynyl-2'-deoxyuridine (EdU) assay results indicated that RAP-LPs@ESCu significantly inhibited the proliferation of GBM cells (Figure S9A-D).

Apoptotic cells undergo sequential membrane changes, starting with phosphatidylserine (PS) externalization (Annexin V⁺/PI⁻) in early apoptosis, followed by complete membrane permeabilization (Annexin V⁺/PI⁺) in late stages 35. In contrast, cuproptosis is driven by mitochondrial copper-dependent protein aggregation, and induces proteotoxic stress without early PS exposure 36, 37. Flow cytometry analysis using Annexin V/PI staining showed that RAP-LPs@ESCu treatment led to the predominant accumulation of cuproptosis-induced GBM cells in Q1 (rapid death), indicating early membrane permeabilization before PS exposure, while apoptotic cells were distributed in Q2 (late apoptosis) and Q3 (early apoptosis) (Figure 2E-F). This quadrant distribution, together with the caspase-independent execution, highlights the mechanistic divergence between cuproptosis and apoptosis. Furthermore, transwell invasion assays showed that the RAP-LPs@ESCu group exhibited the most significant inhibition of cell invasion compared to other groups (Figure 2G) (Figure S10A-B). Collectively, these data indicate that RAP-LPs@ESCu are effective in inducing the death and inhibiting the proliferation of GBM cells* in vitro*.

### RAP-LPs@ESCu induced cuproptosis in GBM cells

**Figure 3:**
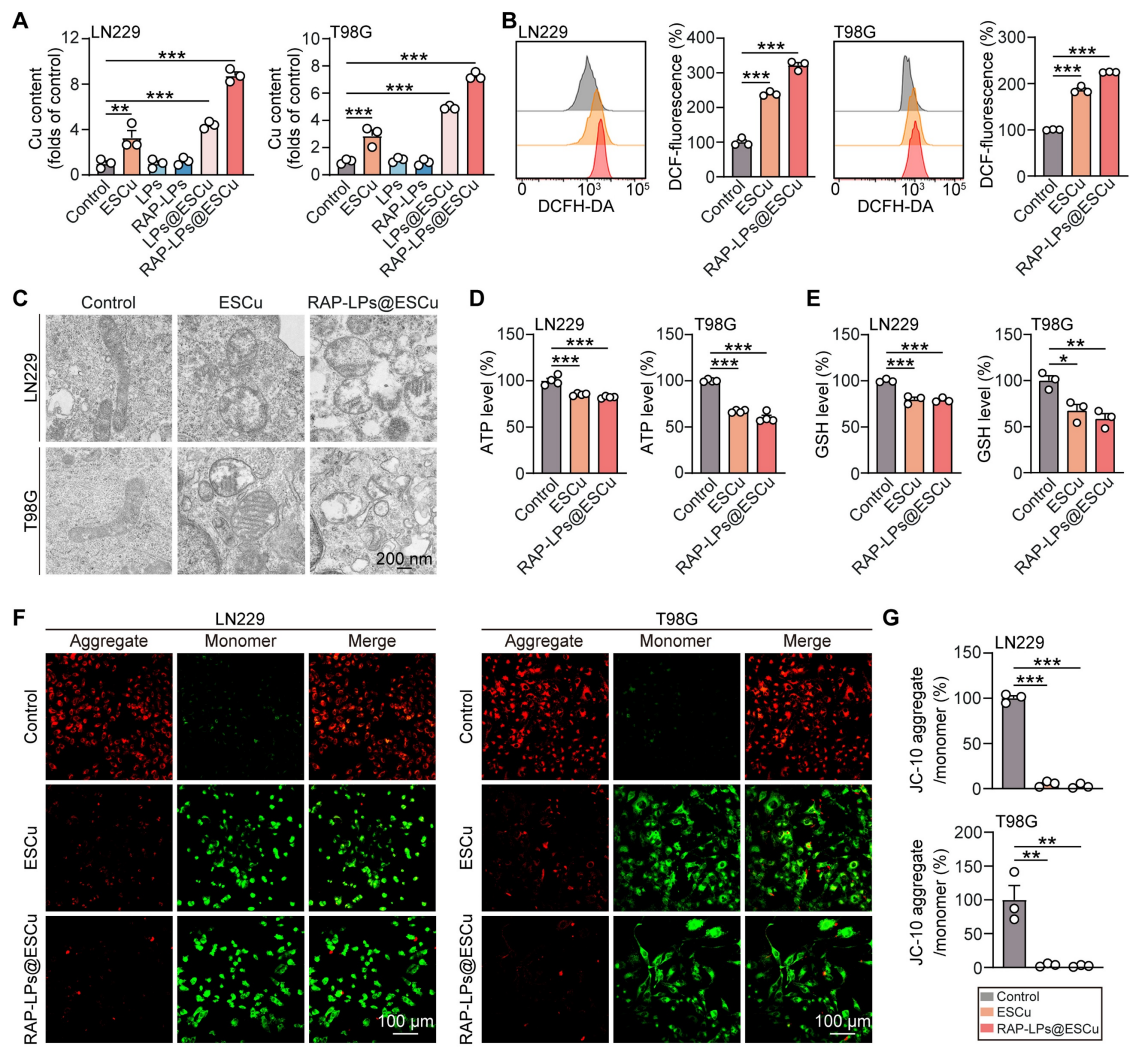
** Effects of RAP-LPs@ESCu on oxidative stress and mitochondrial dysfunction of LN229 and T98G cells.** (A) Copper levels of LN229 and T98G cells measured by AAS after treatment with ESCu, LPs, RAP-LPs, LPs@ESCu, and RAP-LPs@ESCu. (B) Intracellular ROS levels in LN229 and T98G cells assessed via flow cytometry after treatment with ESCu and RAP-LPs@ESCu for 24 h. (C) Representative TEM images of mitochondrial morphology in LN229 and T98G cells treated with ESCu and RAP-LPs@ESCu. Scale bar, 200 nm. (D) ATP levels in LN229 and T98G cells treated with ESCu and RAP-LPs@ESCu. (E) GSH levels in LN229 and T98G cells treated with ESCu and RAP-LPs@ESCu. (F-G) Alterations in MMP (Δ ψ m) treated with ESCu and RAP-LPs@ESCu in LN229 and T98G cells. Scale bar, 100 μm. *n* = 3 independent biological replicates, 3 images were counted per group in each independent trial. *P < 0.05, **P < 0.01, ***P < 0.001.

After cellular internalization, Cu²⁺ dissociates from the ESCu complex and is reduced to the more toxic Cu⁺, while the released ES facilitates further extracellular Cu²⁺ uptake via membrane transport mechanisms 29, 35. Copper overload directly disrupts cellular redox homeostasis by depleting GSH, the primary endogenous antioxidant that normally chelates excess copper ions and mitigates metal-induced oxidative stress (OS) 38. In addition, copper ions induce aberrant lipid acylation of DLAT in cuproptosis, leading to disruption of the tricarboxylic acid (TCA) cycle, reduced ATP production and mitochondrial damage 39-41. To investigate the effects of increased copper levels, we measured copper concentrations in LN229 and T98G cells treated with RAP-LPs@ESCu using AAS, which effectively quantifies copper levels 42. The results indicated that the RAP-LPs@ESCu group had the highest copper levels compared to the other groups (Figure 3A). We then evaluated intracellular ROS using the fluorescent probe DCFH-DA, as described in previous reports 43. Flow cytometry results showed that GBM cells treated with RAP-LPs@ESCu exhibited the highest ROS levels (Figure 3B). Consistently, CLSM analysis revealed the strongest green fluorescence in the RAP-LPs@ESCu group, confirming that RAP-LPs@ESCu induced the greatest ROS production in GBM cells (Figure S11).

Next, mitochondrial function was evaluated. TEM images showed that RAP-LPs@ESCu caused mitochondrial shrinkage, increased membrane density, and decreased mitochondrial cristae in GBM cells (Figure 3C) (Figure S13). JC-10 staining revealed a significant decrease in the ratio of red fluorescent JC-10 aggregates to green fluorescent JC-10 monomers in GBM cells treated with RAP-LPs@ESCu, indicating a reduction in mitochondrial membrane potential (Figure 3F-G). This decrease in mitochondrial function resulted in significantly lower ATP levels in RAP-LPs@ESCu treated cells compared to other groups (Figure 3D). Additionally, our study showed that RAP-LPs@ESCu significantly reduced GSH levels, which may further contribute to the occurrence of cuproptosis (Figure 3E). Mitochondria are the main source of ROS. MitoSOX Red staining further confirmed that ESCu, LPs@ESCu and RAP-LPs@ESCu treatments increased mitochondrial ROS production, which would lead to excessive cellular ROS (Figure S12A-B). These findings collectively demonstrated that RAP-LPs@ESCu triggers cuproptosis through copper dyshomeostasis and mitochondrial impairment‌.

### RAP-LPs@ESCu induced cuproptosis via upregulating FDX1 expression in GBM cells

**Figure 4:**
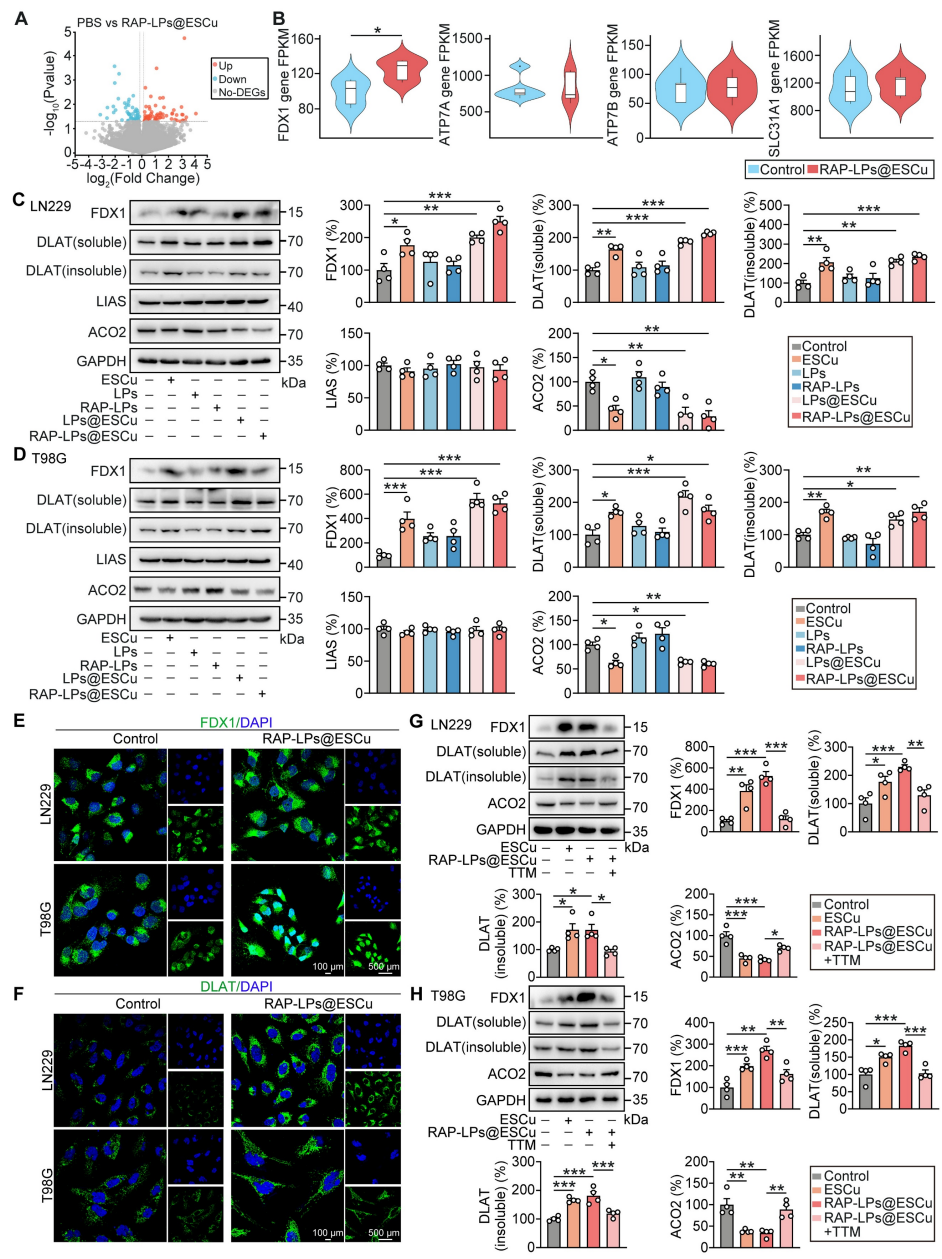
** RAP-LPs@ESCu induced cuproptosis by upregulating FDX1 expression *in vitro*.** (A) Volcano plots of the DEGs between PBS and RAP-LPs@ESCu group. (B) Statistical analysis revealed that RAP-LPs@ESCu significantly increased the expression of the *FDX1* gene in LN229 cells compared to the control group, whereas the mRNA expression of *ATP7A*, *ATP7B*, and *SLC31A1* showed no significant changes. (C-D) Western blot analysis of FDX1, DLAT (soluble), DLAT (insoluble), LIAS and ACO2 protein levels in LN229 and T98G cells treated with ESCu, LPs, RAP-LPs, LPs@ESCu and RAP-LPs@ESCu. *n* = 3 independent biological replicates. (E-F) Immunofluorescence analysis of levels of FDX1 and DLAT in LN229 and T98G cells treated with RAP-LPs@ESCu. Scale bars, 100 μm and 500 μm. (G-H) The protein levels of FDX1, DLAT (soluble), DLAT (insoluble) and ACO2 were detected by Western blot in LN229 and T98G cells treated with various treatments. *n* = 3 independent biological replicates. *P < 0.05, **P < 0.01, ***P < 0.001.

To investigate the molecular mechanism underlying the induction of cuproptosis by RAP-LPs@ESCu, we performed transcriptome RNA-seq on LN229 cells. A total of 134 DEGs were identified between the control and RAP-LPs@ESCu groups (Figure 4A). Kyoto Encyclopedia of Genes and Genomes (KEGG) pathway analysis showed that these target genes were enriched in the pathways regulating cell peroxisome function, neurotransmission and oncogenic signaling (Figure S14). In particular, this study focused on key genes involved in copper metabolism and cuproptosis induction, including* FDX1*, copper-transporting ATPases α (*ATP7A*) and β (*ATP7B*), and solute carrier family 31 member 1 (*SLC31A1*) 44. FDX1 facilitates the reduction of Cu²⁺ to the more cytotoxic Cu⁺ and serves as a critical regulator of cell death, while SLC31A1 facilitates copper transport into cells 38, 45. Importantly, this mechanism operates downstream of copper accumulation and is distinct from the classical copper toxicity pathway regulated by transporters such as ATP7A/B 45. ATP7A and ATP7B are responsible for the export of excess copper, thereby maintaining copper homeostasis 46. Although alterations in their expression can affect cuproptosis, it is highly likely that cuproptosis is induced by activation of downstream execution mechanisms (FDX1 and lipoylated proteins) 47. Notably, our results revealed that *FDX1* expression was significantly upregulated in the RAP-LPs@ESCu group compared to the control group, while no significant changes in the mRNA expression levels of *ATP7A*, *ATP7B* and *SLC31A1* were observed (Figure 4B). Moreover, transcription factors such as *MTF1, SP1, TCF4* and *RUNX1* can regulate the expression of ATP7A, ATP7B and SCL31A1 48-51. However, their expression was not significantly altered, as shown by the RNA-seq data (Figure S15). The above results indicating that the elevated intracellular copper ion levels mediated by RAP-LPs@ESCu were not affected by the expression of copper transport proteins in the cells themselves. In addition, this study found that knockdown of FDX1 in GBM cells significantly increased cell viability in the RAP-LPs@ESCu-treated group. However, treatment with the apoptosis inhibitor Z-VAD and the ferroptosis inhibitor Fer-1 failed to reverse the decreased cell viability in the RAP-LPs@ESCu-treated group. These results suggest that RAP-LPs@ESCu induces cuproptosis in GBM cells, rather than apoptosis or ferroptosis (Figure S16A-B). Collectively, the RAP-LPs@ESCu-induced cuproptosis in GBM cells is modulated by the delivery of copper ions directly into the cell and the upregulation of FDX1.

FDX1-mediated synthesis of Cu^+^ induces the aggregation of lipoylated DLAT and destabilizes of Fe-S cluster proteins, triggering cuproptosis 52. Lipoic acid synthetase (LIAS), a key enzyme in lipoic acid synthesis, catalyzes the incorporation of free sulfur atoms into mitochondrial disulfide cofactors for DLAT lipoylation, and the accumulation of insoluble lipoylated DLAT disrupts mitochondrial function, induces oxidative stress, impairs the TCA cycle, and leads to energy metabolism imbalance and cell death 37, 53. Aconitase 2 (ACO2), an Fe-S cluster protein, is reduced by ES and affected by elevated Cu^+^ levels, which trigger the degradation of proteins and reduce the stability of Fe-S clusters in ACO2 37. These effects led to mitochondrial dysfunction, impaired TCA cycle activity, and ultimately induced cuproptosis 40. The present study showed that RAP-LPs@ESCu significantly increased the expression of FDX1, DLAT (soluble) and DLAT (insoluble), while ACO2 expression decreased in both LN229 and T98G cells (Figure 4C-D), suggesting that RAP-LPs@ESCu induces cuproptosis through promoting the aggregation of lipoylated proteins and the loss of Fe-S cluster proteins. Although previous studies have established that copper promotes cuproptosis by inhibiting LIAS, this effect was not observed in our study 37. Immunofluorescence results further demonstrated increased aggregation of FDX1 and DLAT in cells treated with RAP-LPs@ESCu (Figure 4E-F). Moreover, to further confirm the role of Cu^2+^, GBM cells were treated with the copper ion chelator TTM. The results showed that TTM led to a reduction in the expression of FDX1, DLAT (soluble), and DLAT (insoluble), while ACO2 expression was elevated in LN229 and T98G cells (Figure 4G-H). These findings further support the notion that RAP-LPs@ESCu induced alterations in the protein expression of FDX1, DLAT, and ACO2 through an increase in Cu content.

Taken together, these results suggest that RAP-LPs@ESCu induced cuproptosis in GBM cells by promoting the upregulation of FDX1, which subsequently triggered the aggregation of lipoylated DLAT and the loss of the Fe-S cluster protein ACO2.

### RAP enhanced cellular uptake and BBB penetration of liposomes

**Figure 5:**
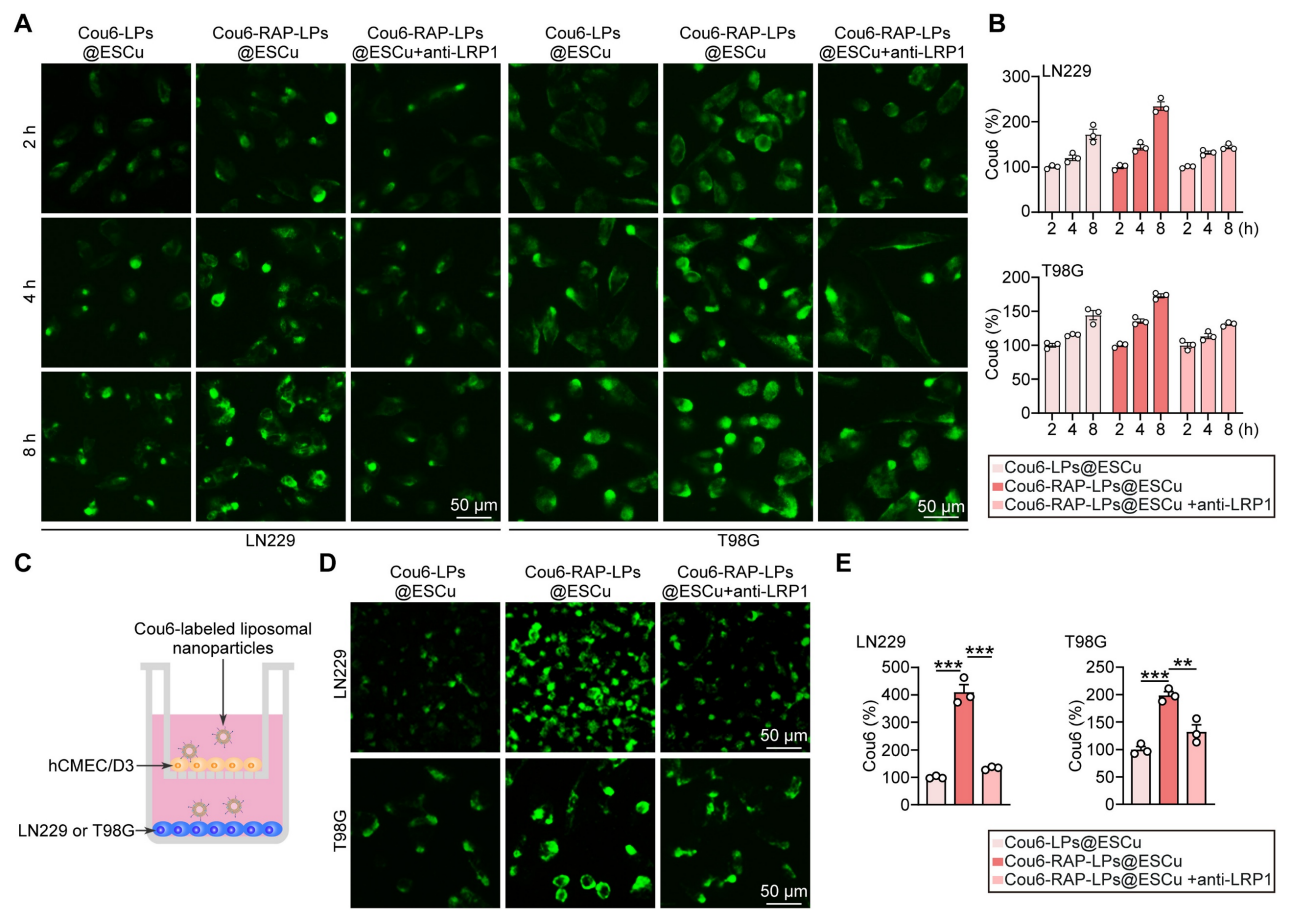
***In vitro* BBB transcytosis of RAP-LPs@ESCu.** (A-B) Representative images illustrating fluorescent signal intensity detected LPs@ESCu and RAP-LPs@ESCu uptake into LN229 and T98G cells for different times. Scale bar, 50 μm. (C) Schematic representation of the *in vitro* BBB model utilizing a transwell system to evaluate the penetration capability of RAP-modified liposomes across the endothelial monolayer. (D-E) Representative images showing fluorescent signal intensity in LN229 and T98G cells following 24 h of treatment with Cou6-labeled liposomes. *n* = 3 independent biological replicates, 3 images were counted per group in each independent trial. **P < 0.01, ***P < 0.001.

Effective cellular internalization is a critical determinant for improving the accumulation of therapeutic agents in tumor cells, a prerequisite for successful cancer treatment 54. Previous studies have shown that RAP facilitates nanoparticle internalization through LRP1 10, 55. To evaluate this mechanism in GBM, we exposed LN229 and T98G cell lines to Cou6-labeled liposomes and quantified intracellular fluorescence accumulation at sequential time points. To further validate the pivotal role of RAP-mediated LRP1 interaction, parallel experiments were performed using anti-LRP1 antibody pre-treatment 56. Fluorescent images showed a time-dependent fluorescence intensification across all groups, with RAP-LPs@ESCu exhibiting significantly superior accumulation compared to control formulations, which was inhibited by the pre-treatment with LRP1 antibody (Figure 5A-B). This finding confirms both the effective cellular uptake of liposomal carriers and the critical enhancement conferred by RAP functionalization.

To further investigate the BBB-penetrating capability of RAP-LPs@ESCu, we employed an *in vitro* BBB model using a transwell assay system. This model was constructed with human brain microvascular endothelial cells (hCMEC/D3), a well-characterized cell line that recapitulates critical BBB features, including tight junction formation and efflux transporter activity 57. Initial cytotoxicity assessment revealed that 400 nM RAP-LPs@ESCu did not induce significant cell death or viability reduction in hCMEC/D3 cells (Figure S17). This finding suggested that the observed BBB penetration was mediated by receptor-specific mechanisms rather than nonspecific barrier disruption through cytotoxicity. To quantitatively evaluate trans-BBB transport efficiency, we performed fluorescence tracking experiments using Cou6-labeled LPs@ESCu and RAP-LPs@ESCu. The optimized *in vitro* BBB model consisted of hCMEC/D3 monolayers in upper chambers and GBM cells in lower chambers, mimicking the physiological endothelial-tumor interface (Figure 5C). After 24 h incubation with Cou6-labeled liposomes, we observed significantly stronger fluorescence signals in GBM cells treated with RAP-LPs@ESCu compared to non-targeted LPs@ESCu. Notably, this enhancement was significantly attenuated by co-treatment with anti-LRP1 antibodies, confirming LRP1 receptor-mediated transcytosis as the predominant transport mechanism (Figure 5D-E).

### Biodistribution and anti-GBM efficacy of RAP-LPs@ESCu

**Figure 6:**
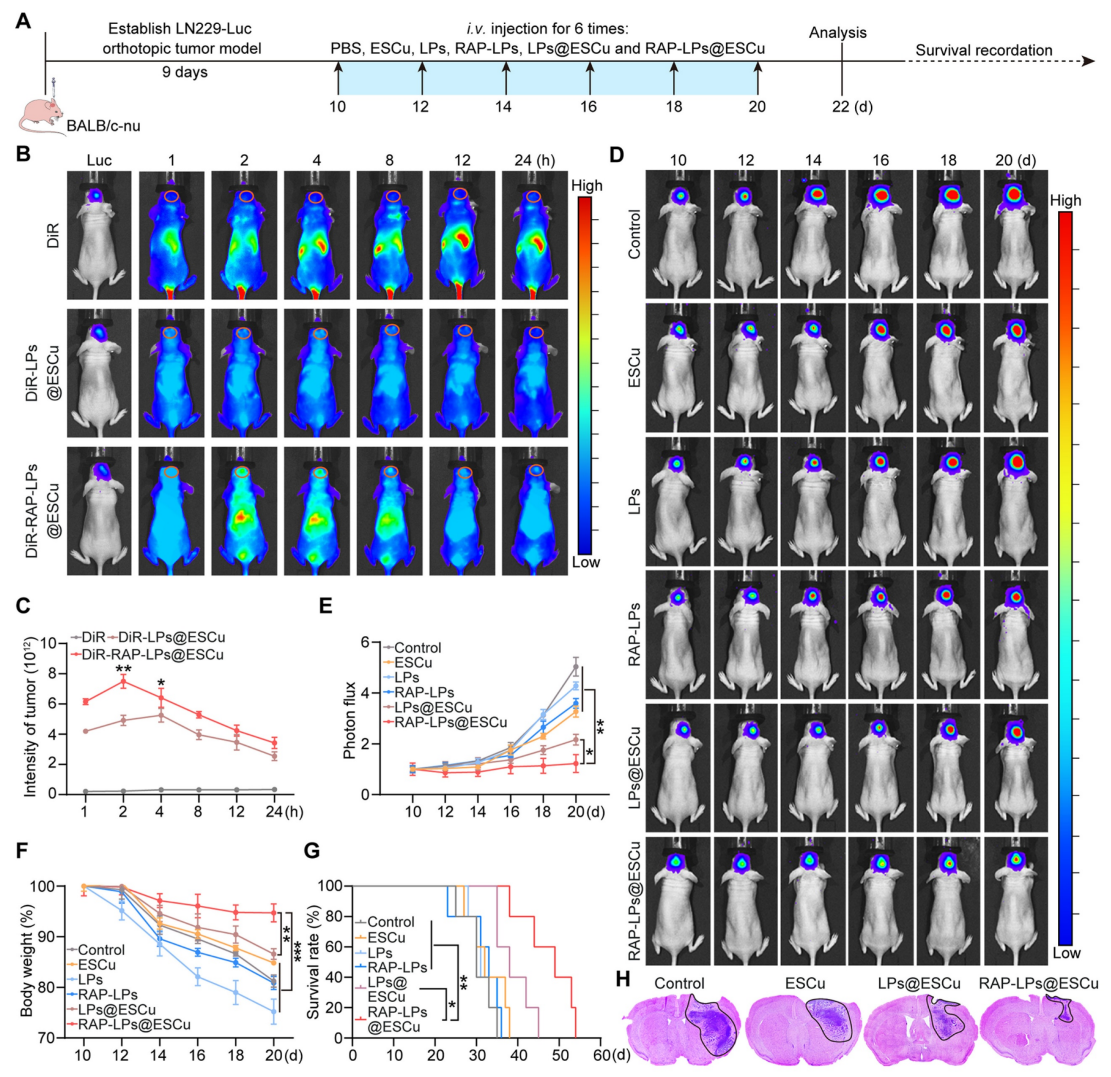
** RAP-LPs@ESCu are recruited to the brain and mediate an anti-GBM effect.** (A) Schematic image of experimental timeline. (B-C) Fluorescence images of orthotopic LN229-bearing nude mice following the injection of DiR-labeled liposomes. *n* = 3 mice. (D-E) Luciferase luminescence levels in mice following treatment with PBS, ESCu, LPs, RAP-LPs, LPs@ESCu and RAP-LPs@ESCu. *n* = 3 mice. (F) Changes in mouse body weights. *n* = 5 mice. (G) Survival curves of LN229-bearing mice exposed to the indicated treatments. *n* = 5 mice. (H) Representative images of H&E staining following treatment with PBS, ESCu, LPs@ESCu and RAP-LPs@ESCu. *n* = 3 mice. *P < 0.05, **P < 0.01, ***P < 0.001.

Based on established evidence that RAP conjugation enhances nanocarrier targeting through ligand-receptor interactions 23, we systematically evaluated the *in vivo* biodistribution and therapeutic potential of RAP-LPs@ESCu in an orthotopic GBM model. LN229-Luc-bearing BALB/c nude mice received intravenous injections of DiR-labelled LPs@ESCu or RAP-LPs@ESCu, with real-time fluorescence imaging quantifying spatio-temporal distribution kinetics. Time-course analysis revealed that RAP-LPs@ESCu reached peak intracranial fluorescence intensity at 2 h post-injection, whereas non-targeted LPs@ESCu required 4 h to reach maximum tumor accumulation (Figure 6B-C). This acceleration of BBB penetration highlights the critical role of RAP-mediated active transport in overcoming physiological barriers. *Ex vivo* organ biodistribution showed that the fluorescence accumulation in the free DiR group was mainly concentrated in the liver and spleen after 24 h injection, whereas the fluorescence signal in the DiR-RAP-LPs@ESCu group was stronger at the tumor site, suggesting that the off-target accumulation in the peripheral organs (heart, liver, spleen, lungs, and kidneys) was minimal in the RAP-LPs@ESCu-treated mice, confirming enhanced tumor selectivity and reduced reticuloendothelial system clearance. (Figure S18A-B).

Quantitative Cu biodistribution studies using ICP-OES precisely revealed the GBM targeting properties of RAP-LPs@ESCu* in vivo*. Biodistribution results indicated predominant liver Cu accumulation; however, in GBM-bearing mice treated with RAP-LPs@ESCu, Cu content significantly increased to 19.93% ID/g, markedly higher than in ESCu (1.03% ID/g) and LPs@ESCu (1.81% ID/g) treated groups (Figure S19A). Pharmacokinetic analysis demonstrated a slow decline in blood Cu concentration in RAP-LPs@ESCu-treated mice, maintaining relatively high levels (4.03 ± 0.02 μg/mL) after 24 h compared to free ESCu (0.65 ± 0.01 μg/mL) and LPs@ESCu groups (0.80 ± 0.01 μg/mL) (Figure S19B). Additionally, free ESCu underwent rapid renal clearance, while liposomal encapsulation delayed Cu release. Notably, urinary Cu concentration in the RAP-LPs@ESCu group (2.48% ± 0.01 μg/mL) was significantly higher than in the ESCu group (0.30% ± 0.01 μg/mL) but lower than in the LPs@ESCu group (4.11% ± 0.03 μg/mL) (Figure S19C), suggesting preferential GBM accumulation with delayed urinary excretion. Collectively, these findings demonstrate that RAP-LPs@ESCu enhances GBM targeting, prolongs the *in vivo* circulation of ESCu, and improves its bioavailability.

To establish the derived effects of enhanced tumor targeting, we conducted comprehensive therapeutic evaluations in orthotopic GBM models (experimental timeline: Figure 6A). IVIS was employed to assess tumor growth and showed that RAP-LPs@ESCu significantly delayed the progression of GBM (Figure 6D-E). Importantly, this potent anti-tumor activity correlated with improved physiological parameters. Mice in the RAP-LPs@ESCu treatment group showed only a slight decrease in body weight, while the other groups exhibited significant weight loss (Figure 6F). Survival analysis further confirmed that RAP-LPs@ESCu significantly prolonged the lifespan of the mice (Figure 6G). Histopathological validation via H&E staining showed reduction in tumor cross-sectional area with RAP-LPs@ESCu versus other groups (Figure 6H). To comprehensively evaluate the neuroprotective effects of RAP-LPs@ESCu, the neurological deficit and motor impairment scoring, along with the open field test, were employed to evaluate the neurological function and motor abilities of the mice 58, 59. The RAP-LPs@ESCu-treated group showed a significant reduction in scores compared to other groups (Figure S22A). Furthermore, open field testing revealed increased total distance traveled in the RAP-LPs@ESCu-treated group, suggesting enhanced locomotor ability (Figure S22B-C). Moreover, given the critical importance of biosafety in the development of nanocarrier systems for GBM therapy, we conducted a rigorous biosafety profiling of RAP-LPs@ESCu, to address key concerns regarding hemocompatibility and systemic toxicity of nanocarrier. Routine blood counts and serum biochemical analysis showed that the levels of red blood cells (RBCs), white blood cells (WBCs), platelets (PLTs), total bile acids (TBAs), alanine aminotransferase (ALT), aspartate aminotransferase (AST), total bilirubin (TBIL), and direct bilirubin (DBIL) were within the safe range across all treatment groups (Figure S23A-H). The hemolysis assays demonstrated that the concentration of ESCu used in this study had negligible toxicity to erythrocytes (Figure S24). Furthermore, H&E staining revealed no significant pathological changes in major organs (heart, liver, spleen, lung and kidney) after different treatments (Figure S23I). These results supported the biosafety of RAP-LPs@ESCu for the treatment of GBM.

### RAP-LPs@ESCu induced cuproptosis by upregulating FDX1 in GBM tissue

**Figure 7:**
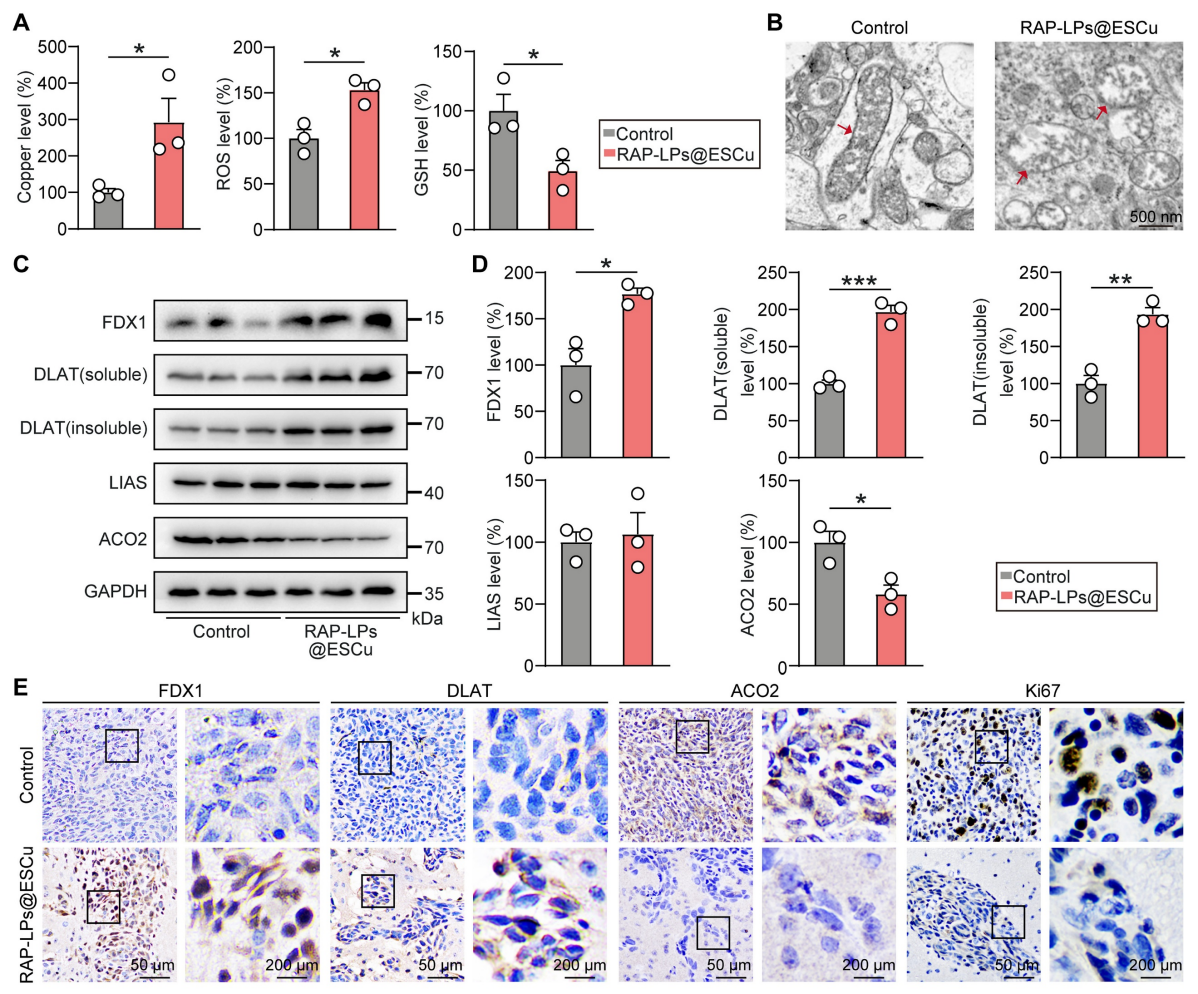
** Anti-GBM efficacy of RAP-LPs@ESCu induced cuproptosis.** (A) Copper content, ROS levels and GSH levels in GBM tissues treated with RAP-LPs@ESCu. (B) Representative TEM images of mitochondria. Scale bar, 500 nm. (C-D) Western blot was used to detect the protein expression of FDX1, DLAT (soluble) DLAT (insoluble), ACO2 and LIAS* in situ* GBM tissues. (E) Representative immunohistochemistry images of FDX1, DLAT, ACO2 and Ki67 in GBM. *n* = 3 mice. Scale bars, 50 μm and 200 μm. *P < 0.05, **P < 0.01, ***P < 0.001.

The *in vivo* anti-GBM mechanism of RAP-LPs@ESCu was validated through the cuproptosis-dependent cell death pathway mediated by FDX1. In orthotopic LN229-Luc models, RAP-LPs@ESCu treatment triggered significant intratumoral copper accumulation, accompanied by GSH depletion and ROS increase, thereby establishing a pro-cuproptotic redox milieu (Figure 7A) (Figure S20). Ultrastructural analysis revealed hallmark mitochondrial perturbations, including cristae fragmentation and outer membrane rupture, consistent with copper-induced proteotoxic stress (Figure 7B). Mechanistically, Western blot showed that RAP-LPs@ESCu upregulated FDX1 expression and promoted DLAT oligomerization (both soluble and insoluble DLAT were significantly increased), while downregulating ACO2, thereby disrupting Fe-S cluster biogenesis, with no significant change observed for LIAS (Figure 7C-D) (Figure S21A-B). Immunohistochemical analysis revealed a significant increase in FDX1 and DLAT levels and a suppression of ACO2 specifically within tumor regions (Figure 7E) (Figure S21C). Ki67 is a widely recognized biomarker that was analyzed in this study to assess cell proliferation 42. The concomitant reduction in Ki67-positive cells, consistent with *in vitro* EdU assays, confirms that cuproptosis inhibits cell proliferation within the GBM tissues (Figure 7E). These results demonstrated that RAP-LPs@ESCu orchestrate tumor-selective copper overload, FDX1-driven lipoylation crisis, and ultimately execution of cuproptosis *in vivo*.

## Conclusion

**Figure 8:**
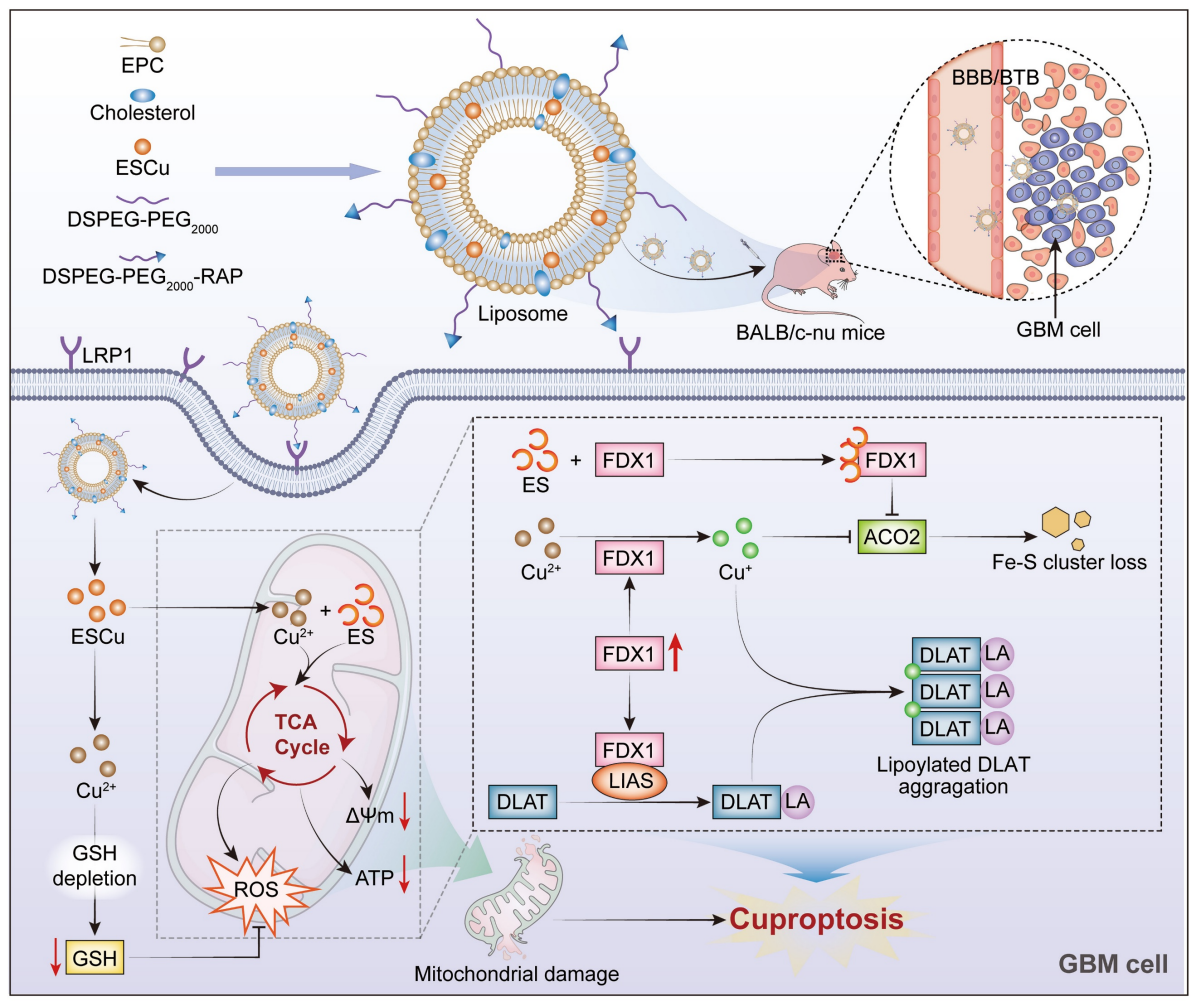
** Schematic representation of RAP-LPs@ESCu-induced cuproptosis for GBM suppression.** RAP-LPs@ESCu, prepared by film hydration, penetrated the BBB and BTB into GBM cells via RAP-LRP1-mediated cellular uptake. RAP-LPs@ESCu upregulated FDX1 expression in GBM cells. Binding of FDX1 to LIAS promoted lipoylation modification of DLAT. Cu^2+^ released from RAP-LPs@ESCu was converted to Cu^+^ by FDX1. Subsequently, Cu^+^ aggregates lipoylated DLAT, contributing to toxicity. In addition, Cu^2+^ and Cu^+^ depleted intracellular GSH levels and promoted the loss of the Fe-S cluster protein ACO2. Together, these effects resulted in impaired mitochondrial function, ultimately triggering cuproptosis in GBM cells.

To summarize, this study successfully constructed RAP-LPs@ESCu, a targeted liposomal formulation encapsulating ESCu. RAP-LPs@ESCu demonstrated a favorable biosafety profile and effectively delivered ESCu to GBM cells in both *in vitro* and *in vivo* studies. This resulted in an increase in intracellular Cu^2+^ levels, upregulation of FDX1 expression, subsequent induction of cuproptosis, and ultimately resulting in tumor growth inhibition (Figure 8). In conclusion, RAP-LPs@ESCu shows promising potential as an effective treatment strategy for GBM.

## Supplementary Material

Supplementary methods and figures.

## Abbreviations

AAS: atomic absorption spectroscopy
ACO2: aconitase 2
AST: aspartate aminotransferase
ATP7A: copper-transporting ATPases α
ATP7B: copper-transporting ATPases β
BBB: blood-brain barrier
BTB: blood-tumor barrier
CLSM: confocal laser scanning microscope
DBIL: direct bilirubin
DLAT: dihydrolipoamide S-acetyltransferase
DLS: dynamic light scatting
EdU: 5-ethynyl-2'-deoxyuridine
EE%: encapsulation efficiency
ES: elesclomol
ESCu: elesclomol-copper complex
EPC: egg yolk lecithin
FCM: flow cytometry
FDX1: ferredoxin-1
Fe-S: iron-sulfur
FESEM: field emission scanning electron microscopy
GBM: glioblastoma
GSH: glutathione
hCMEC/D3: human cerebral microvascular endothelial cells
IC50: half-maximal inhibitory concentration
ICP-OES: inductively coupled plasma optical emission spectrometry
IVIS: *in vivo* imaging system
LIAS: lipoic acid
LPs: liposomes
LRP1: low-density lipoprotein receptor-related protein-1
LN229-Luc: luciferase-labeled LN229
OS: oxidative stress
PI: propidium iodide
PEG: polyethylene glycol
PI: propidium Iodide
PLTs: platelets
RAP: receptor-associated protein
RBCs: red blood cells
ROIs: regions of interest
ROS: reactive oxygen species
SLC31A1: solute carrier family 31 member 1
TBAs: total bile acids
TBIL: total bilirubin
TCA: tricarboxylic acid
TEM: transmission electron microscopy
TTM: tetrathiomolybdate
XPS: x-ray photoelectron spectroscopy
