# Supplementary material for: Engineered RAP-anchored copper-escorting liposomes for FDX1-targeted cuproptosis in glioblastoma therapy‌
Source: Theranostics. 2025 Jul 2;15(15):7802–19. doi: 10.7150/thno.115723 (PMC12316038; doi:10.7150/thno.115723)
Supplement: Supplementary file 1 — Supplementary methods and figures. [file thnov15p7802s1.pdf]

Methods

SDS-PAGE with Coomassie blue staining

SDS-PAGE was performed to confirm the successful loading of RAP onto liposomes. Briefly, samples including LPs, RAP-LPs, LPs@ESCu, RAP-LPs@ESCu, and free RAP (as the positive control) were heated in loading buffer and then subjected to SDS-PAGE electrophoresis at a constant voltage of 100 V for 4 h. The gel was subsequently collected and stained with Coomassie blue, destained with a destaining solution, and finally observed and imaged.

In vitro release

The *in vitro* release profiles of ESCu from RAP-LPs@ESCu at different pH were evaluated using dialysis (MW 3500). Briefly, liposomes (1 mL) were placed into a dialysis bag, which was then immersed in a 37 °C PBS buffer solution (pH 5.0 and pH 7.4). Subsequently, 1 mL of the solution was withdrawn for ESCu determination and replaced with an equal amount of fresh PBS solution.

Cell proliferation inhibition assay

The EdU imaging kit (K1175, AEPxBIO) was used to assess the proliferative capacity of GBM cells. LN229 and T98G cells were seeded in 24-well plates with slides. After treatments, the EdU stock solution was diluted in complete medium, and half of the cell culture medium was replaced to achieve a final EdU concentration of 10 μM. Following a 2 h incubation, the medium was discarded, and cells were fixed with 4% paraformaldehyde for 15 min. Cells were then permeabilized with 0.5% Triton X-100 at room temperature for 25 min. The permeabilizing solution was discarded, and a click chemistry reaction was performed using EdU reaction buffer, $CuSO_4$, 6-FAM azide, and EdU buffer additive for 30 min protected from light. After the reaction, the nuclei were stained with Hoechst 33342 solution. EdU-labeled cells were observed under a confocal laser scanning microscope (CLSM) (A1, Nikon), with positive cells appearing green and nuclei blue.

Cell culture and treatment

LN229 and T98G cells were cultured in DMEM supplemented with 10% fetal bovine serum and 1% penicillin/streptomycin at 37 °C with 5% CO_2. For FDX1 knockdown, cells were transfected with siFDX1. In addition, cells were treated with RAP-LPs@ESCu (400 nM, equivalent ESCu concentration 100 nM), Z-VAD (50 μM,

HY-164388, Med Chem Express) and Ferrostatin-1 (Fer-1) (2 μM, HY-100579, Med Chem Express) for 24 h.

***In vivo* pharmacokinetic study**

The orthotopic LN229-bearing nude mice were randomly assigned to 3 groups receiving ESCu, LPs@ESCu and RAP-LPs@ESCu, respectively. Blood and urine samples were collected at predetermined time points (1, 2, 4, 8, 12 and 24 h for blood; 6, 12 and 24 h for urine) for Cu concentration measurement using inductively coupled plasma optical emission spectrometry (ICP-OES).

Assessments of neurological impairments and movement disorders

Neurologic deficit and motor impairment score was used to evaluate the neuroprotective effect of RAP-LPs@ESCu. The evaluation was conducted using the Longa 5-point scale, i.e. 0, no detectable neurological deficits; 1, inability to fully extend the left forelimb; 2, spontaneous circling to the left while walking; 3, leaning to the right during locomotion; 4, inability to walk independently; and 5, mortality.

Similarly, the locomotor capacity of mice was assessed using the open field test. Mice in each group were individually placed in an open field reaction chamber (50 × 50 × 50 cm) for 5 min, and the total distance travelled was recorded by SMART 3.0 software.

***In vivo* biosafety**

Following all treatments, blood samples were collected from the mice for the analysis of routine hematological parameters, including white blood cells (WBCs), red blood cells (RBCs), platelets (PLTs), as well as biochemical markers such as total bilirubin (TBIL), direct bilirubin (DBIL), aminotransferase (ALT), and aspartate transaminase (AST).

Major organs (heart, liver, spleen, lungs, and kidneys) were collected and fixed in 4% paraformaldehyde for paraffin embedding and subsequent hematoxylin and eosin (H&E) staining. Briefly, tissue sections were deparaffinized, rehydrated, and stained with hematoxylin for 5 min. After differentiation with acid-ethanol, the sections were stained with eosin for 3 min. Finally, the sections were dehydrated through graded alcohols and mounted with neutral balsam for observation.

Hemolysis assay

Hemolysis of LPs, RAP-LPs, LPs@ESCu, and RAP-LPs@ESCu was assessed across various concentration of ESCu (0, 5, 10, 20, 50 100, 200 μg/mL). Mouse blood

was collected and centrifuged at 1000 g for 20 min to obtain the erythrocytes, which were then washed and diluted with PBS. The liposome PBS dispersion was prepared by mixing 800 μL of liposome preparations with 200 μL of diluted erythrocytes. PBS served as the negative control (0% hemolysis), while distilled water was used as the positive control (100% hemolysis). The samples were incubated at 37 °C for 4 h. After 4 h, the samples were centrifuged at 1000 g for 15 min, and the absorbance at 540 nm was measured using a UV/VIS spectrophotometer. Hemolysis (%) less than 5% were considered nontoxic. Percent hemolysis was calculated as follows:

$$\text{Hemolysis (\%)} = (A_{sample} - A_{negative}) / (A_{positive} - A_{negative}) \times 100\%$$

A_{sample} is the absorbance of samples; $A_{positive}$ is the absorbance value of positive controls; $A_{negative}$ is the absorbance value of negative controls.

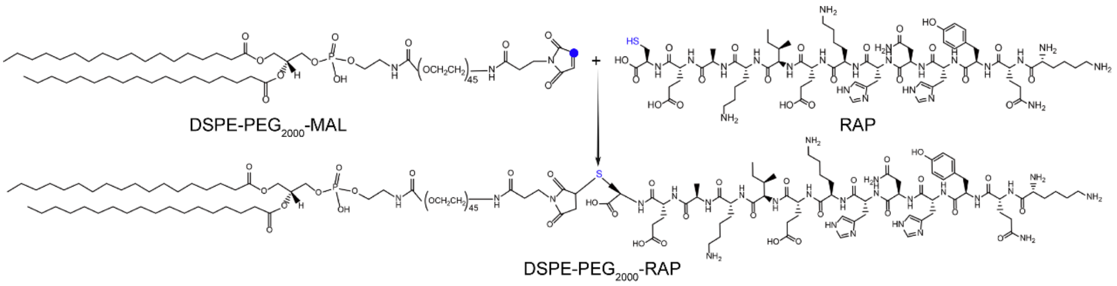

Figure S1 Schematic diagram of DSPE-PEG$_{2000}$-RAP preparation. DSPE-PEG$_{2000}$-RAP prepared by Michael's addition reactions between DSPE-PEG$_{2000}$-MAL and RAP peptides.

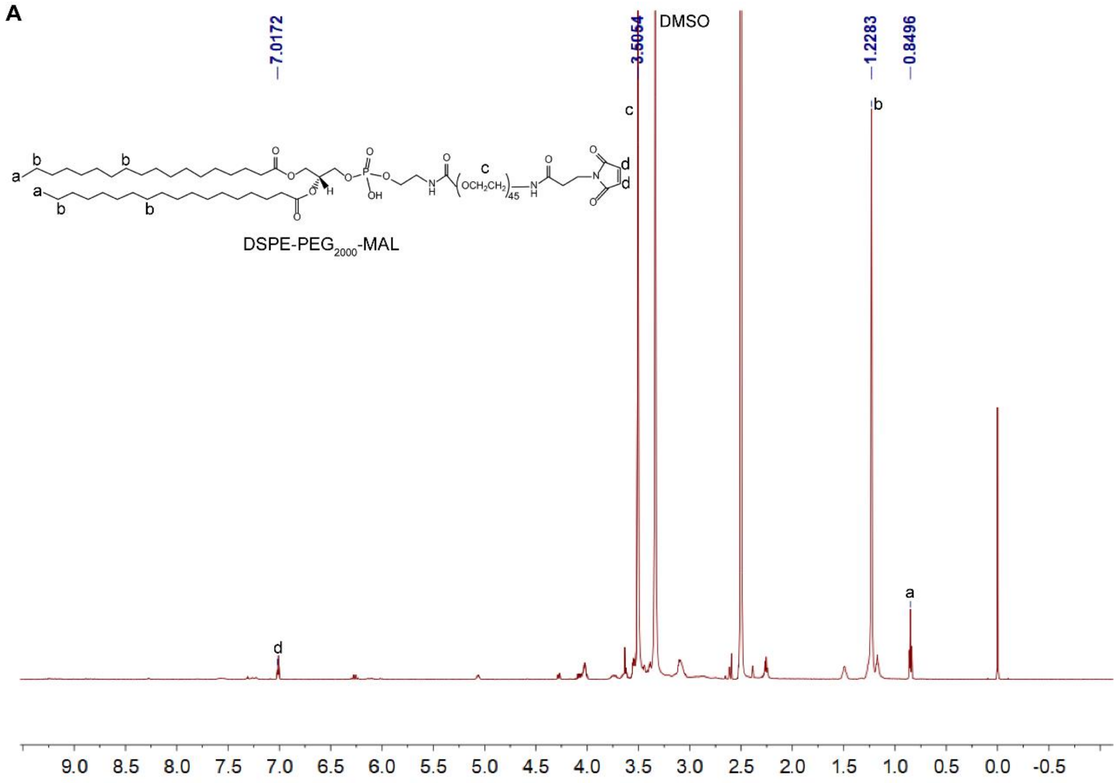

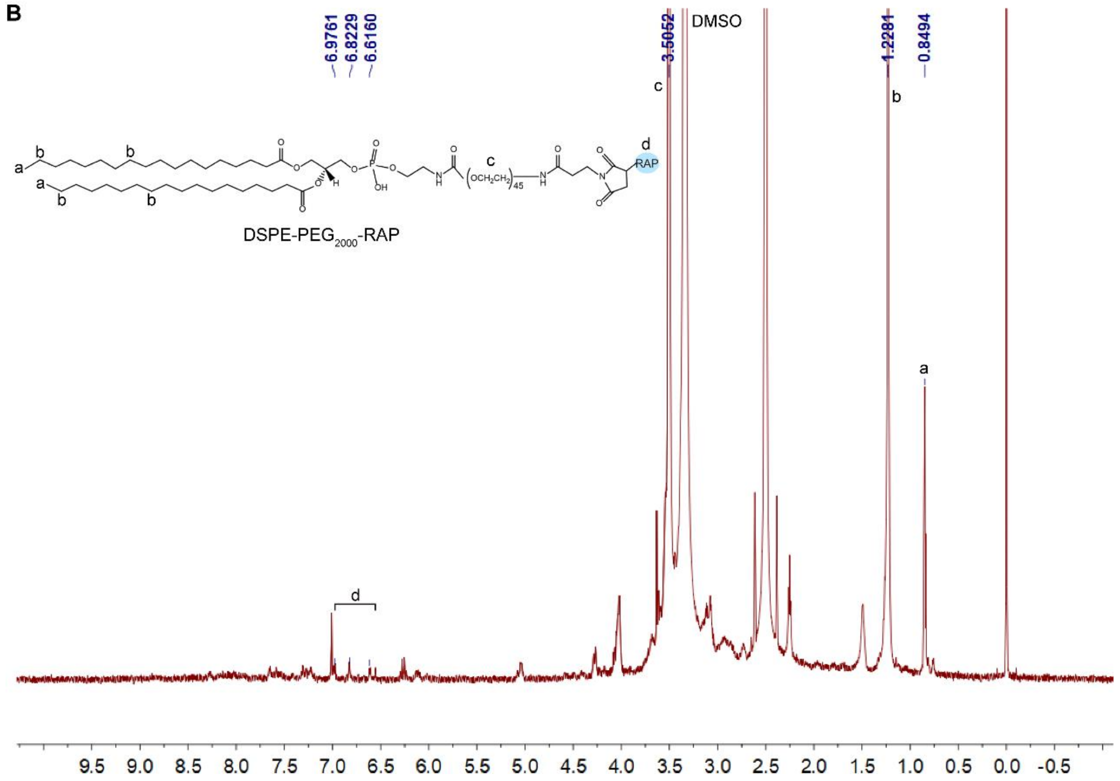

Figure S2 Characterization of DSPE-PEG$_{2000}$-RAP. (A-B) ^{1}H NMR spectra of DSPE-PEG$_{2000}$-MAL and DSPE-PEG$_{2000}$-RAP.

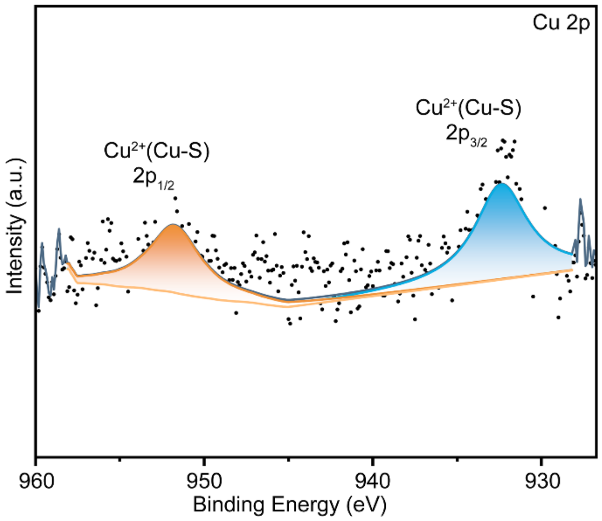

Figure S3 XPS spectra of ESCu. High-resolution Cu 2p.

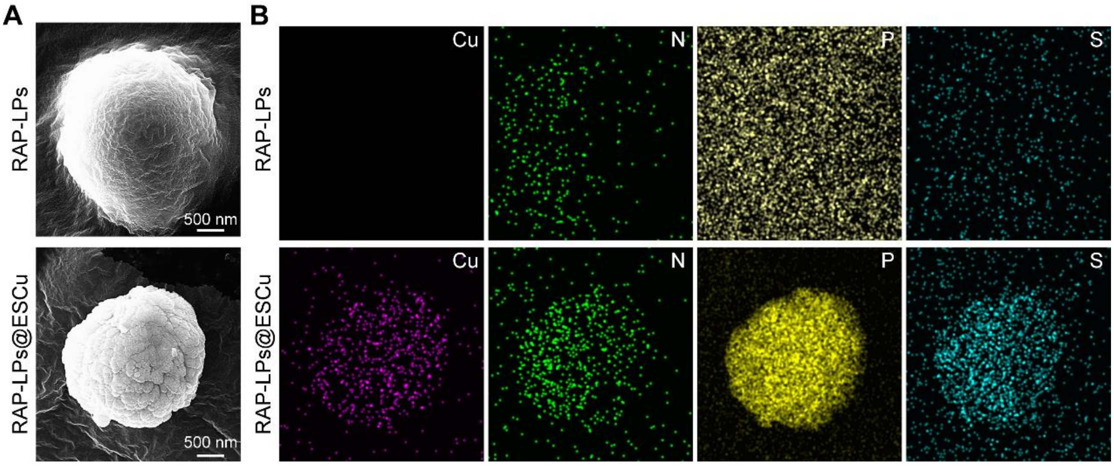

Figure S4 Characterization of RAP-LPs and RAP-LPs@ESCu. (A) Representative SEM images of RAP-LPs and RAP-LPs@ESCu. Scale bar, 500 nm. (B) Representative element maps (Cu, N, P and S) of RAP-LPs and RAP-LPs@ESCu. ESCu formula: $C_{19}H_{18}CuN_4O_2S_2$.

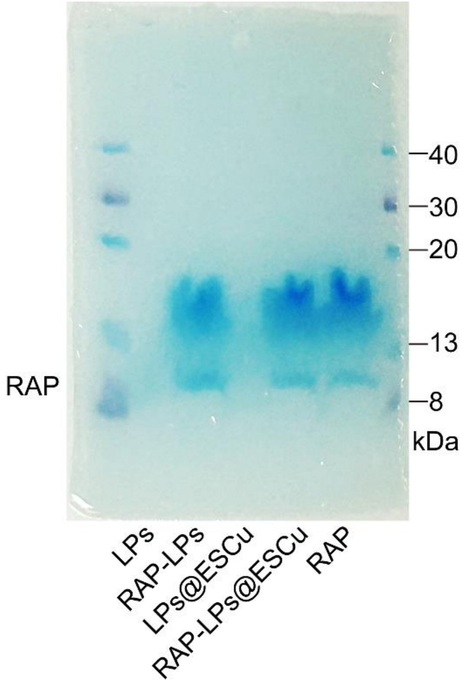

Figure S5 Characterization of RAP-LPs@ESCu. SDS-PAGE analysis of RAP conjugation to the LPs.

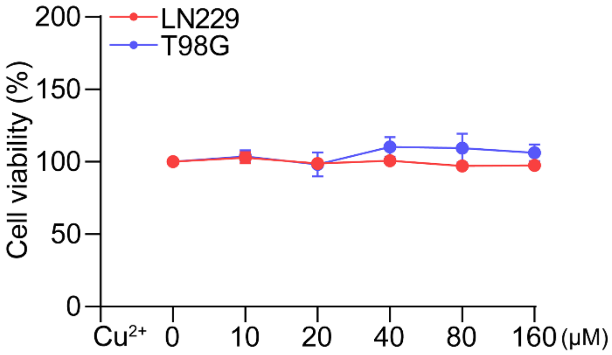

Figure S6 Cell viability of LN229 and T98G cells with Cu^{2+}. Cu^{2+} in the range of 0 to 160 μM was not significantly toxic to cells.

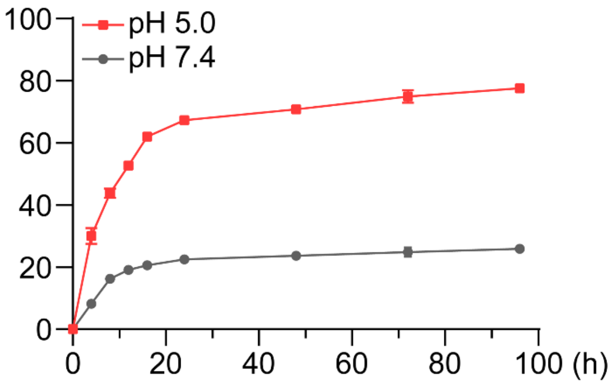

Figure S7 *In vitro* cumulative release profiles of RAP-LPs@ESCu at pH 5.0 and

pH7.4 condition. The cumulative release of Cu^{2+} from RAP-LPs@ESCu reached 77.563 ± 1.293 % and 25.906 ± 1.316 % at 96 h.

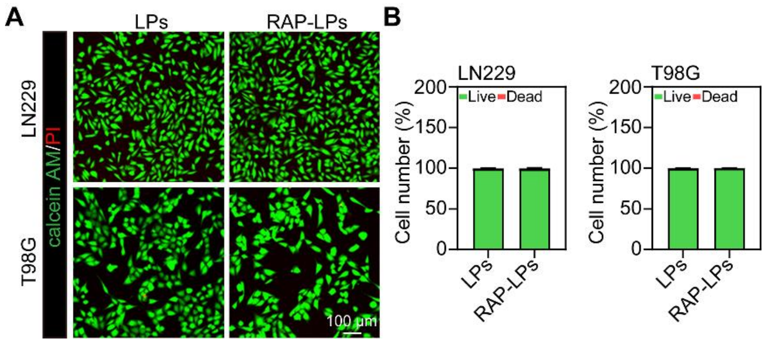

Figure S8 Cell viability stained with calcein-AM and PI. (A-B) Representative images of LN229 and T98G cells treated with LPs and RAP-LPs. Scale bar, 100 μm. *n* = 3 independent biological replicates, 3 images were counted per group in each independent trial.

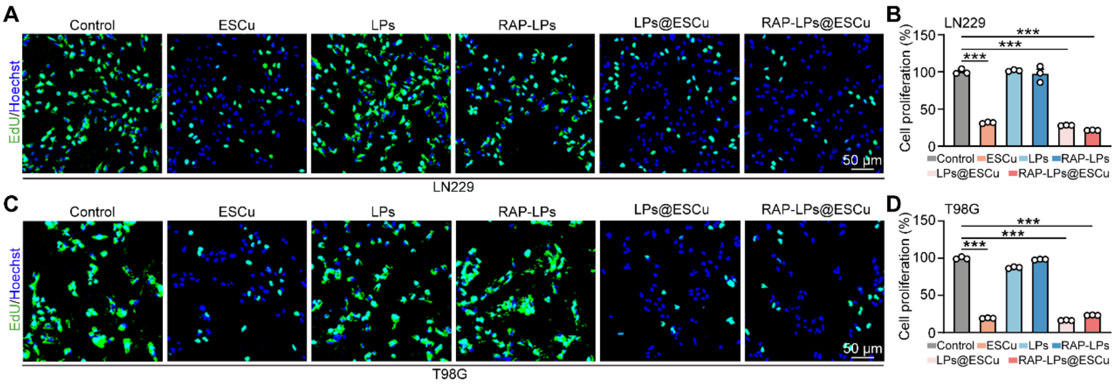

Figure S9 Anti-GBM proliferation of RAP-LPs@ESCu *in vitro*. (A-D) Representative images of EdU/Hoechst staining in LN229 and T98G cells. Scale bar, 50 μm. *n* = 3 independent biological replicates, 3 images were counted per group in each independent trial. ****P* < 0.001.

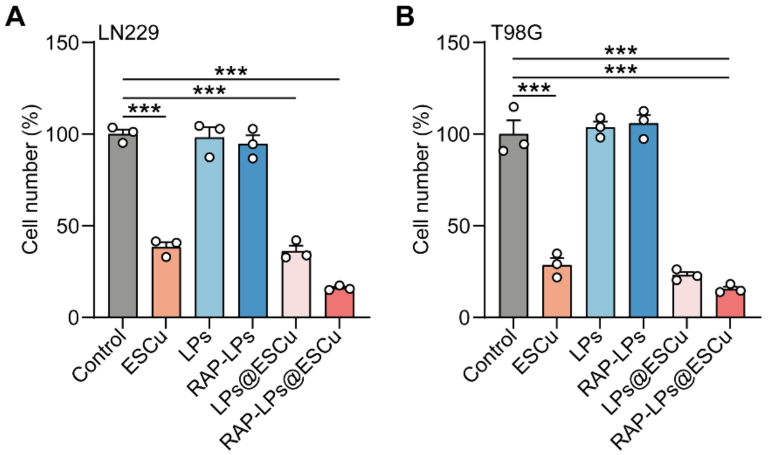

Figure S10 Suppression of GBM cells invasion by RAP-LPs@ESCu. (A-B) Quantitative analysis of invasive capacity in treated LN229 and T98G cells. $n = 3$ independent biological replicates, 3 images were counted per group in each independent trial. ***$P < 0.001$.

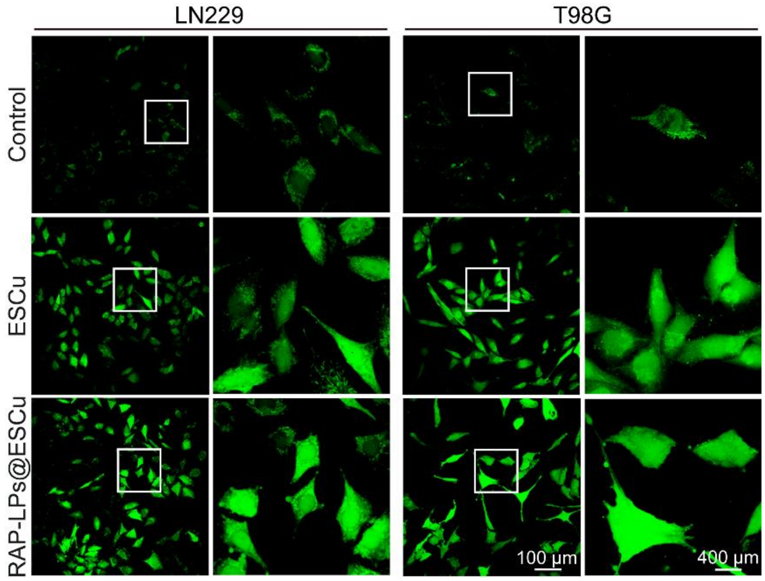

Figure S11 Representative images of DCFH-DA staining in LN229 and T98G cells treated with ESCu and RAP-LPs@ESCu. Scale bars, 100 μm and 400 μm. $n = 3$ independent biological replicates.

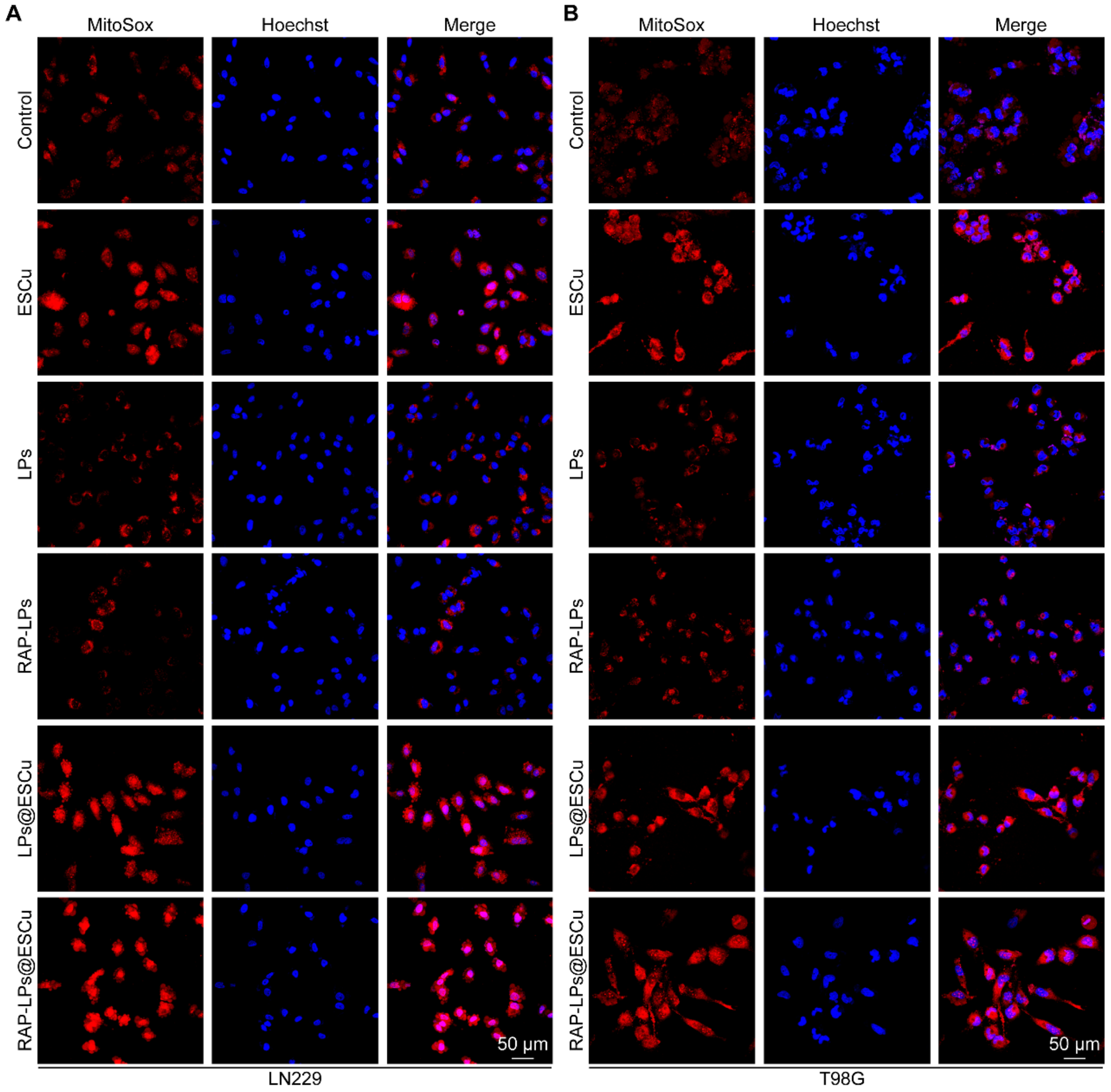

Figure S12 RAP-LPs@ESCu induced mitochondrial oxidative stress. Representative images of MitoSox staining in LN229 and T98G cells treated with ESCu, LPs, RAP-LPs, LPs@ESCu and RAP-LPs@ESCu for 24 h**.** Scale bar, 50 μm. n = 3 independent biological replicates.

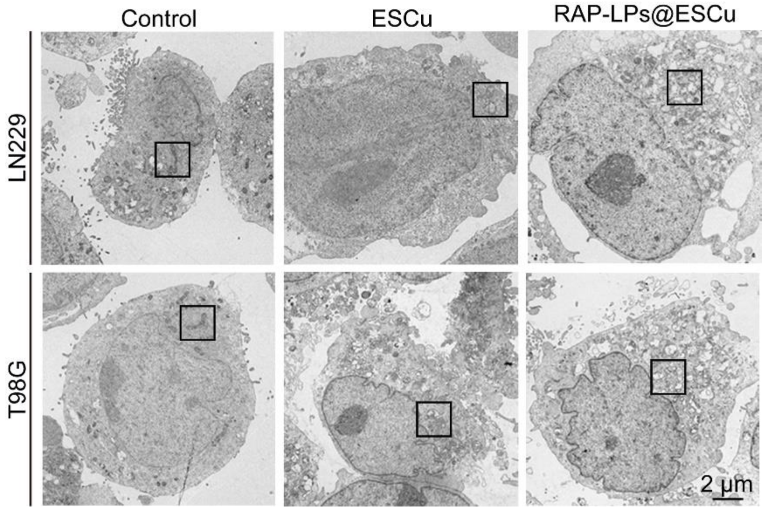

Figure S13 Effects of RAP-LPs@ESCu on oxidative stress and mitochondrial dysfunction of LN229 and T98G cells. The black selection indicates the enlarged area and is shown in Figure 3C. Scale bar, 2 μm.

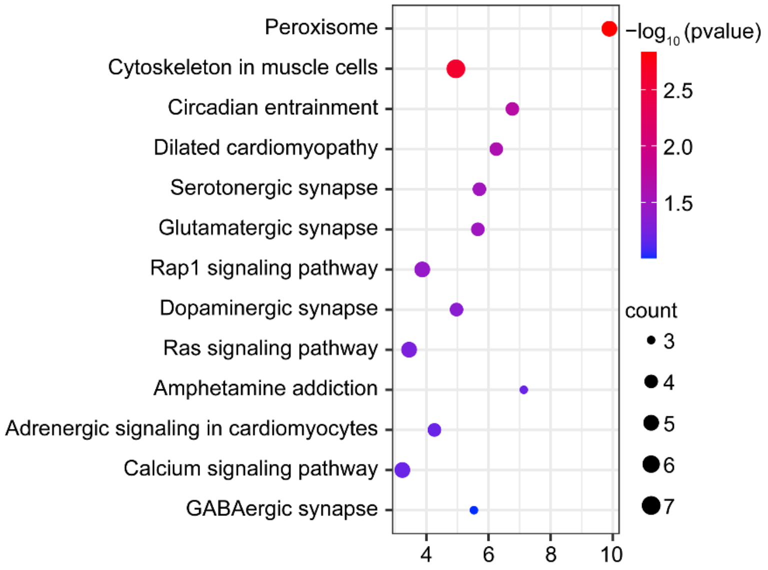

Figure S14 KEGG pathway enrichment analysis. KEGG analysis of DEGs between LN229 cells treated with PBS and RAP-LPs@ESCu.

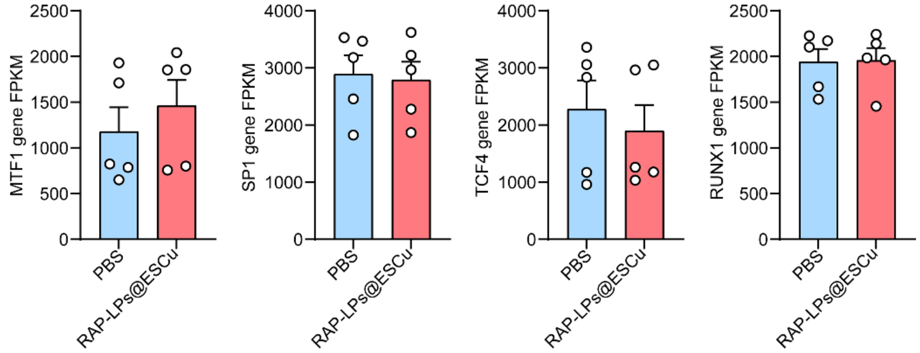

Figure S15 RAP-LPs@ESCu induced cuproptosis by upregulating FDX1 expression *in vitro*. Statistical analysis revealed that the mRNA expression of MTF1, SP1, TCF4, and RUNX1 showed no significant changes between the PBS and RAP-LPs@ESCu groups in LN229 cells.

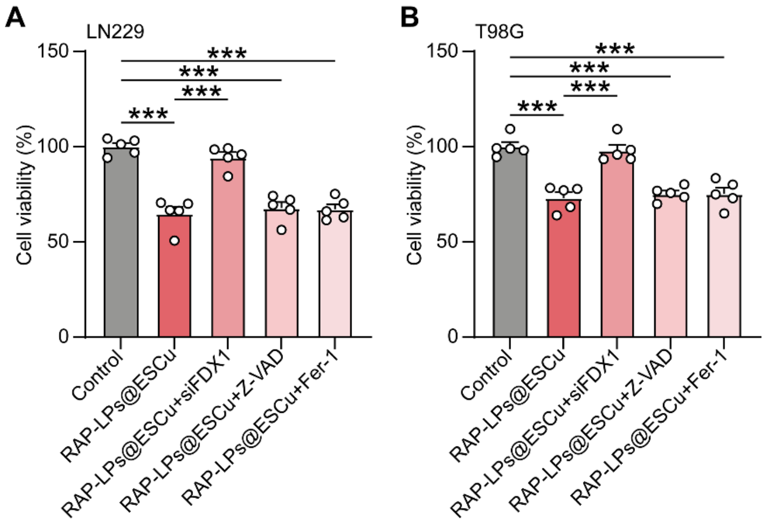

Figure S16 Anti-GBM activity of RAP-LPs@ESCu by downregulating FDX1. (A-B) Cell viability of LN229 and T98G cells with different treatments. $n = 3$ independent biological replicates. $***P < 0.001$.

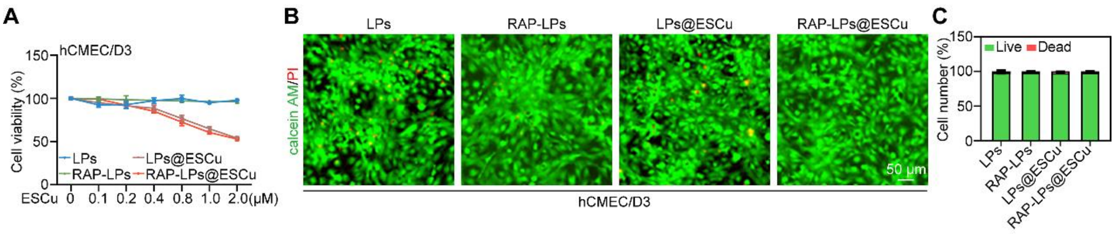

Figure S17 *In vitro* cytotoxicity of RAP-LPs@ESCu on hCMEC/D3 cells. (A) Cytotoxicity of liposomes against hCMEC/D3 cells at various concentrations. (B-C) Representative images of hCMEC/D3 cells exposed to different treatments, stained

with calcein AM and propidium iodide (PI), are presented. Scale bar, 50 μm. n = 3 independent biological replicates, 3 images were counted per group in each independent trial.

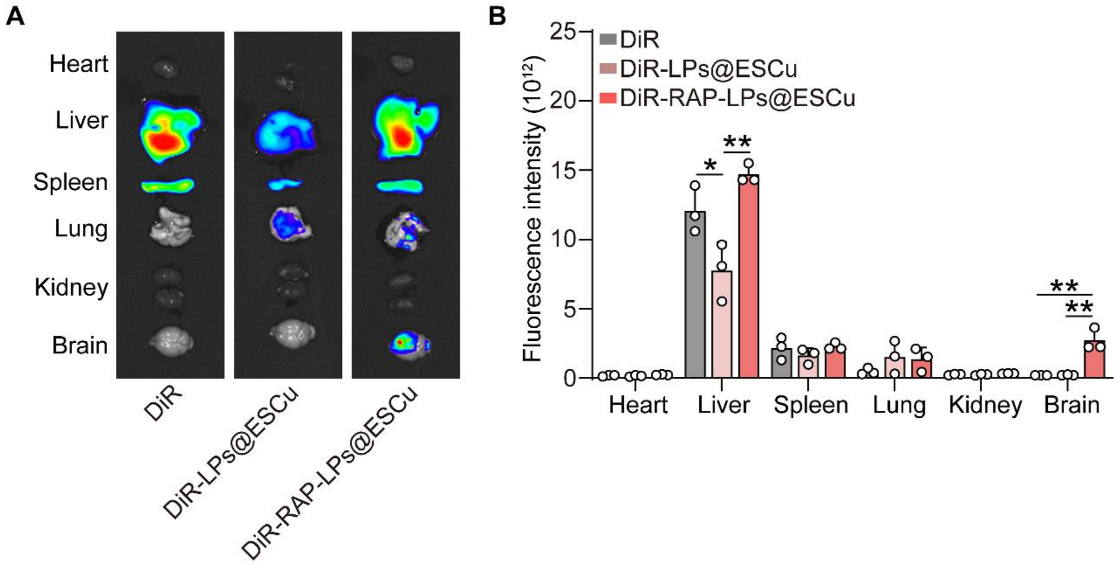

Figure S18 The accumulation of RAP-LPs@ESCu in major organs. (A-B) Fluorescence images of heart, liver, spleen, lung, kidney and brain after 24 h injection. n = 3 mice. *P < 0.05, **P < 0.01.

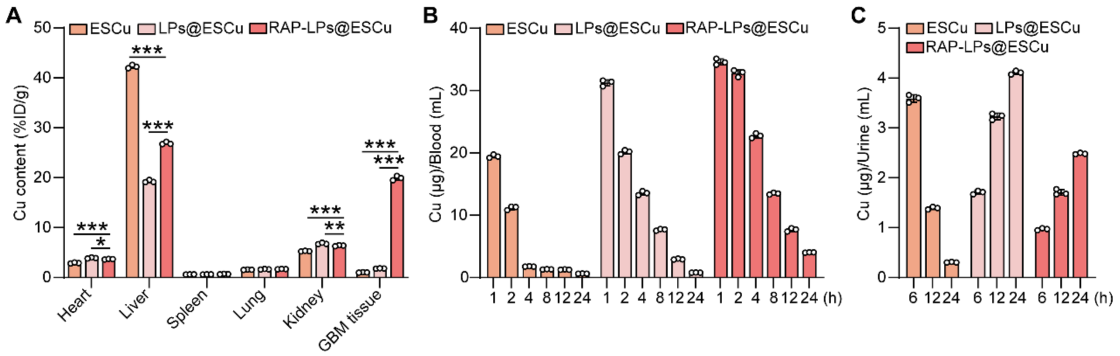

Figure S19 The biodistribution and pharmacokinetics of RAP-LPs@ESCu. (A) The content of Cu (%ID/g) in major organs (heart, liver, spleen, lung and kidney) and GBM tissues at 24 h post-injection was measured by ICP-OES. (B-C) The content of Cu (μg/mL) in blood and urine in situ GBM model mice at different time points via ICP-OES. n = 3 mice. *P < 0.05, **P < 0.01, ***P < 0.001.

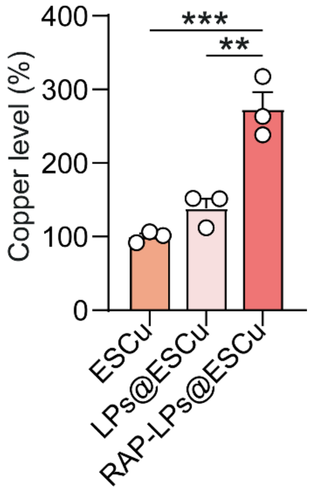

Figure S20 RAP-LPs@ESCu reduced copper level in GBM tissues. The copper content in GBM tissues treated with ESCu, LPs@ESCu and RAP-LPs@ESCu. n = 3 mice. $^{**}P < 0.01$, $^{***}P < 0.001$.

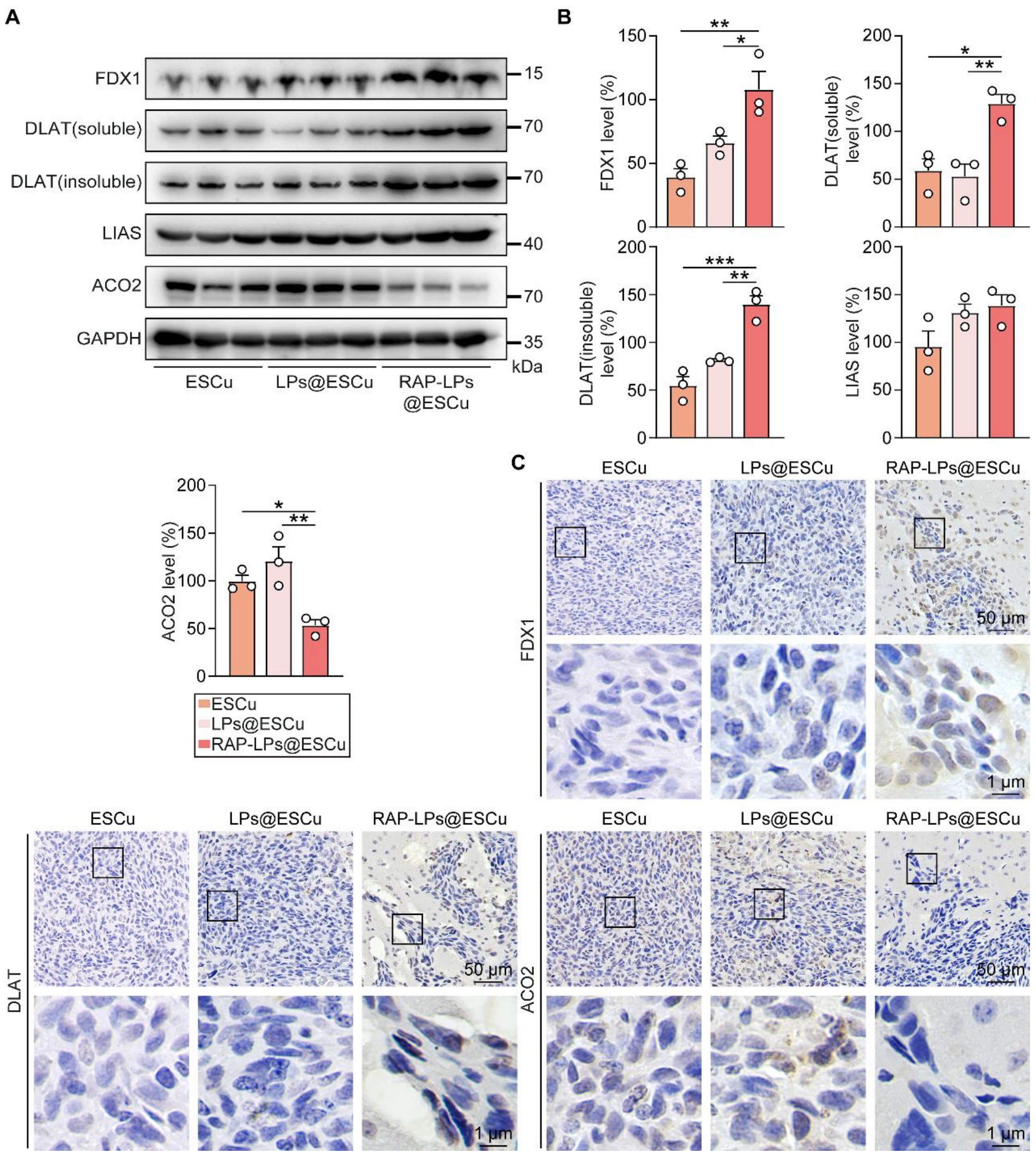

Figure S21 Anti-GBM efficacy of RAP-LPs@ESCu induced cuproptosis. (A-B) Western blot was used to detect the protein expression of FDX1, DLAT (soluble) DLAT (insoluble), ACO2 and LIAS *in situ* GBM tissues. (C) Representative immunohistochemistry images of FDX1, DLAT and ACO2 in GBM. Scale bars, 50 μm and 1 μm. *n* = 3 mice. **P* < 0.05, ***P* < 0.01, ****P* < 0.001.

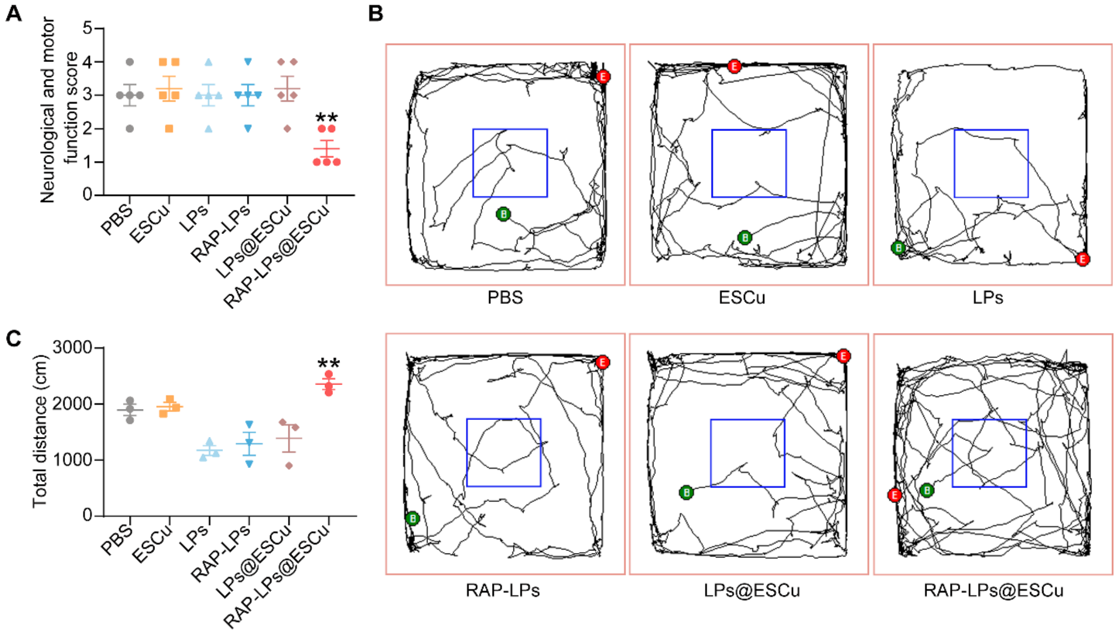

Figure S22 Assessment of neurological and motor functions in LN229-Luc bearing mice. (A) Evaluation of neurological and motor impairments following treatment with PBS, ESCu, LPs, RAP-LPs, LPs@ESCu and RAP-LPs@ESCu. n = 5 mice. (B-C) Representative images of locomotor routes and quantitative analysis of total distance traveled under various treatments. n = 3 mice. **$P < 0.01$.

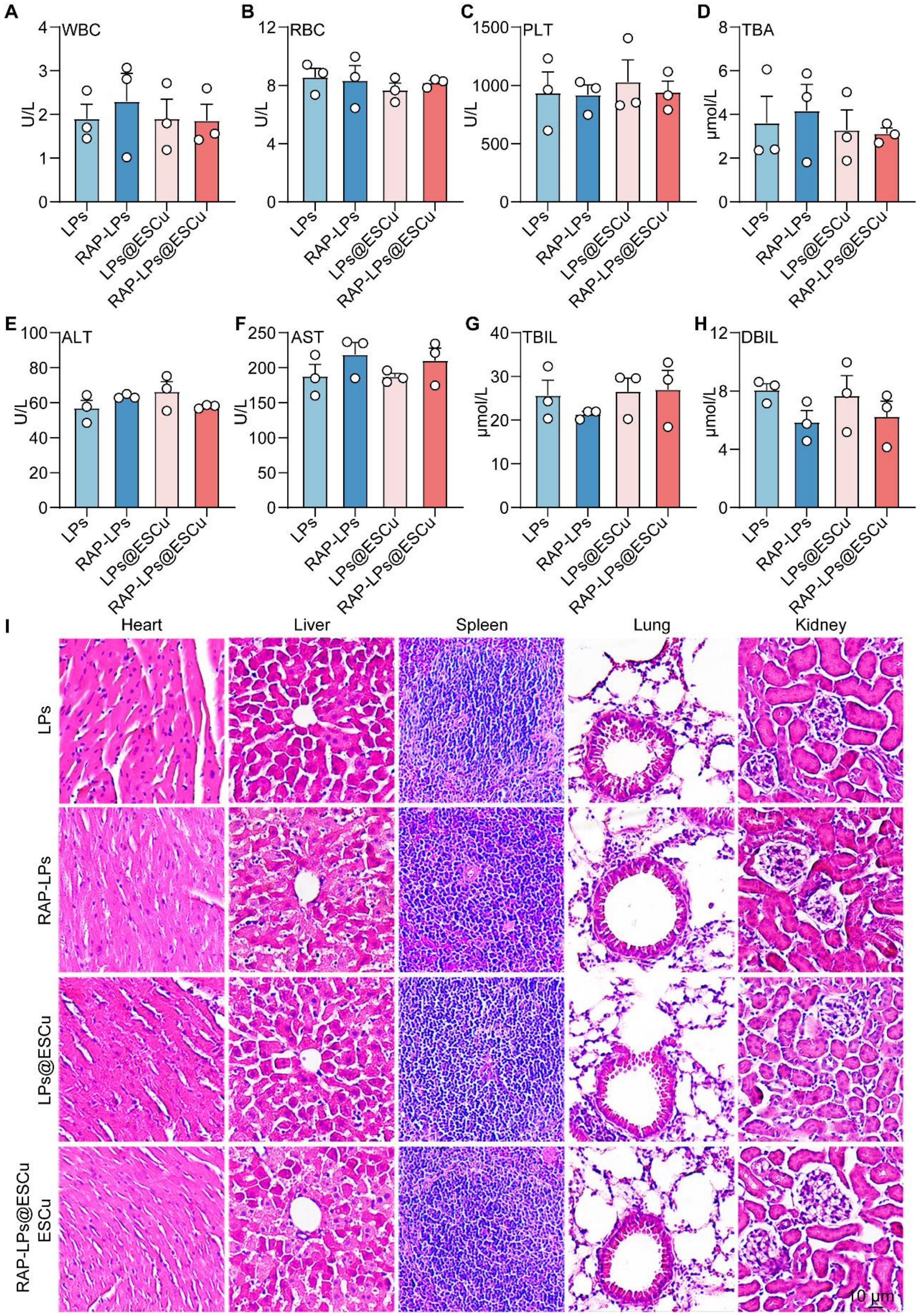

Figure S23 *In vivo* assessment of systemic toxicity. (A-C) Blood routine examination analysis of WBC, RBC and PLT in LN229-Luc bearing nude mice treated with LPs, RAP-LPs, LPs@ESCu and RAP-LPs@ESCu. (D-H) Serum biochemical analysis of TBA, ALT, AST, TBIL and DBIL in LN229-Luc bearing mice treated with LPs, RAP-

LPs, LPs@ESCu and RAP-LPs@ESCu. n = 3 mice. (I) Histological analyses of major organs by H&E staining following different treatments in orthotopic LN229-Luc bearing nude mice. Scale bar, 10 μm. n = 3 mice.

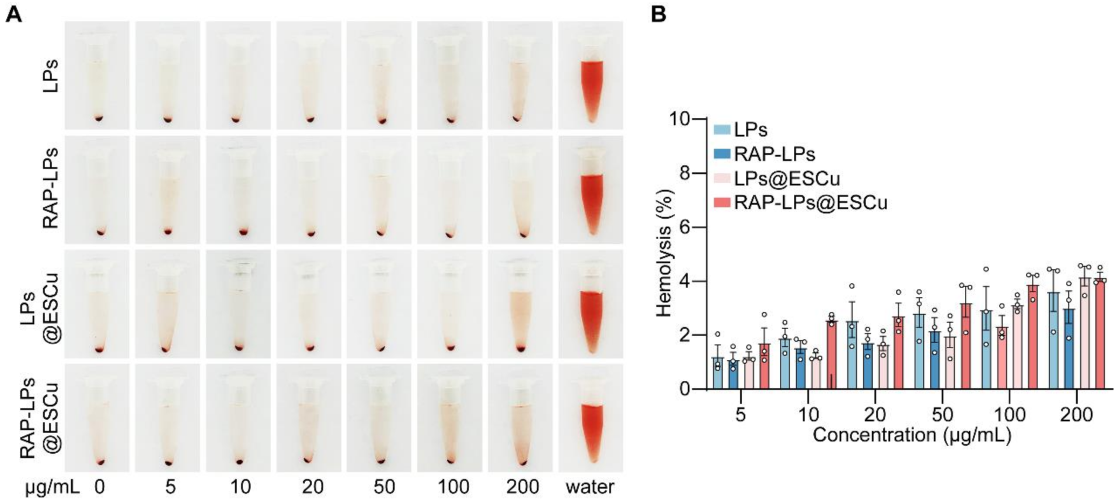

Figure S24 Hemolysis assays of liposomes. (A-B) Representative images of hemolysis after treatment with different concentrations of LPs, RAP-LPs, LPs@ESCu and RAP-LPs@ESCu. n = 3 mice.
